# Hundreds of LncRNAs Display Circadian Rhythmicity in Zebrafish Larvae

**DOI:** 10.3390/cells10113173

**Published:** 2021-11-15

**Authors:** Shital Kumar Mishra, Zhaomin Zhong, Han Wang

**Affiliations:** 1 Center for Circadian Clocks, Soochow University, Suzhou 215123, China; mishrasz@suda.edu.cn (S.K.M.); zhongzhaomin@suda.edu.cn (Z.Z.); 2 School of Biology & Basic Medical Sciences, Medical College, Soochow University, Suzhou 215123, China

**Keywords:** zebrafish larvae, circadian rhythmicity, circadian clocks, noncoding RNAs, lncRNAs, lncRNA-encoded peptides, bioinformatics

## Abstract

Long noncoding RNAs (lncRNAs) have been shown to play crucial roles in various life processes, including circadian rhythms. Although next generation sequencing technologies have facilitated faster profiling of lncRNAs, the resulting datasets require sophisticated computational analyses. In particular, the regulatory roles of lncRNAs in circadian clocks are far from being completely understood. In this study, we conducted RNA-seq-based transcriptome analysis of zebrafish larvae under both constant darkness (DD) and constant light (LL) conditions in a circadian manner, employing state-of-the-art computational approaches to identify approximately 3220 lncRNAs from zebrafish larvae, and then uncovered 269 and 309 lncRNAs displaying circadian rhythmicity under DD and LL conditions, respectively, with 30 of them are coexpressed under both DD and LL conditions. Subsequently, GO, COG, and KEGG pathway enrichment analyses of all these circadianly expressed lncRNAs suggested their potential involvement in numerous biological processes. Comparison of these circadianly expressed zebrafish larval lncRNAs, with rhythmically expressed lncRNAs in the zebrafish pineal gland and zebrafish testis, revealed that nine (DD) and twelve (LL) larval lncRNAs are coexpressed in the zebrafish pineal gland and testis, respectively. Intriguingly, among peptides encoded by these coexpressing circadianly expressed lncRNAs, three peptides (DD) and one peptide (LL) were found to have the known domains from the Protein Data Bank. Further, the conservation analysis of these circadianly expressed zebrafish larval lncRNAs with human and mouse genomes uncovered one lncRNA and four lncRNAs shared by all three species under DD and LL conditions, respectively. We also investigated the conserved lncRNA-encoded peptides and found one peptide under DD condition conserved in these three species and computationally predicted its 3D structure and functions. Our study reveals that hundreds of lncRNAs from zebrafish larvae exhibit circadian rhythmicity and should help set the stage for their further functional studies.

## 1. Introduction

The circadian clock, an endogenous time-keeping mechanism, regulates unique 24-h rhythms of metabolism, physiology and behavior [1]. The suprachiasmatic nucleus (SCN) of the hypothalamus hosts the master clock that drives the circadian rhythms in various tissues and organs [2]. The malfunction of circadian clocks has been closely linked to health problems, such as sleep disorders, mental diseases, and cancers [3]. A variety of model organisms including the fruit fly (*Drosophila melanogaster*) and the zebrafish (*Danio rerio*) [4] have been used to study the operating mechanisms of circadian clocks. The fruit fly is an ideal organism to investigate circadian clocks in insects [5] because of its easy genetic manipulation, breeding in a controlled environment, and monitoring of locomotor activities [6]. The zebrafish is also an attractive organism to study the circadian clock in vertebrates [7,8,9]. The ease of obtaining a large number of zebrafish early embryos enables investigation of the onset of circadian rhythmicity [4]. Recently, the genetic dissection of the zebrafish circadian clock has demonstrated that zebrafish share conserved transcription/translation negative feedback loops with fruit flies, mice and humans [10,11,12]. In particular, zebrafish embryos are transparent and do not require feedings for several days post fertilization [13,14]. Hence the confounding effects of feeding can be avoided [15]. As such, zebrafish embryos/larvae allow for studying circadian rhythms independent of feeding.

lncRNAs represent a diverse set of noncoding RNAs that contain more than 200 nucleotides [16]. Interestingly, several lncRNAs have been implicated in regulating circadian rhythms, including circadian rhythms in cancer cells [17,18]. The expression patterns of nearly 100 lncRNAs were shown to be closely linked to the synthesis of hormone melatonin in the rat pineal gland [19]. Melatonin is an integral component of the circadian clock system [7]. The testis is responsible for several key biological functions, such as producing the germ cells and circulating testosterone, the most active androgen [20]. However, the debate over the existence of circadian rhythms in the testis is still unresolved. For example, some studies suggest a lack of circadian clocks in the testis [21], whereas other studies strongly support the presence of circadian activity in the testis [22]. These findings inspired us to investigate the lncRNA-mediated circadian activities in both the pineal gland and testis [8], thereby uncovering 586 and 165 rhythmically expressed lncRNAs in zebrafish pineal gland and testis, respectively. In particular, 26 rhythmically expressed lncRNAs were shown to be coexpressed in both organs [8]. We hypothesize that some lncRNAs are also rhythmically expressed in zebrafish larvae.

Although lncRNAs do not encode canonical proteins, recent studies suggest that they are involved in numerous fundamental biological processes, including determination of cell fate [23], gene regulation [24], transcription, and various diseases [24]. In fact, thousands of lncRNAs have been identified [25] from a diverse set of organisms, including humans [26,27]. For example, GENCODE v7 contains14,880 human lncRNA transcripts [28], while the ZFLNC lncRNA database [29] catalogues over 21,000 zebrafish lncRNAs. Interestingly, numerous lncRNAs [30] have been shown to encode micropeptides, consisting of approximately 100 amino acids [31]. The micropeptides differ from the functional proteins that often contain more than 400 amino acids [32]. The lncRNA-encoded micropeptides have been demonstrated to regulate various biological processes and activities, such as muscle function, transcription, and mRNA stability [33]. Toddler, a lncRNA-encoded microspeptide, regulates Apelin receptors in order to regulate cell movement in zebrafish [34]. A skeletal muscle-specific lncRNA-encoded micropeptide, myoregulin, (MLN) was found to regulate muscle performance [35]. A recent study [36] discovered a conserved 79-amino acid long microprotein, FORCP, encoded by lncRNA LINC00675. A 60-amino acid long micropeptide ASRPS, encoded by lncRNA LINC00908, contained in small open reading frames [37,38]. A micropeptide, miPEP155, encoded by lncRNA MIR155HG was shown to suppress autoimmune inflammation [30]. In particular, our computational analysis recently revealed hundreds of coding lncRNAs in zebrafish [39].

Despite all the recent progress in understanding lncRNAs, the lncRNA-mediated regulation of circadian rhythms requires further investigation. In particular, additional research is required to elucidate whether the rhythmically expressed lncRNAs are expressed in different organs/tissues, whether some of these coexpressing lncRNAs are conserved in different species, and how light affects rhythmicity of the lncRNAs. Here, we generated RNA-seq-based time-course expression profiles for 3446 unannotated transcripts from wild-type (WT) zebrafish larvae (Supplementary Figure S1A). The datasets were derived from zebrafish larvae at six time points with a 4-h interval under both DD and LL conditions. We computationally analyzed these unannotated transcripts and found 3220 lncRNAs. The rhythmicity analysis of these lncRNAs revealed hundreds of circadian lncRNAs under both DD and LL conditions. We exploited these circadianly expressed lncRNAs to find their potential orthologs from NCBI databases, and used the best matches to perform GO, GOG, and KEGG pathway analyses. We compared these zebrafish larval lncRNAs with those of zebrafish pineal gland and zebrafish testis and revealed nine and twelve lncRNAs coexpressed among larvae, pineal gland and testis under DD and LL conditions, respectively. Subsequently, we assessed the 3D structures and functions of peptides encoded by these coexpressed lncRNAs. The conservation analysis of the zebrafish larval lncRNAs revealed one and four orthologs shared by zebrafish, mice, and humans under DD and LL conditions, respectively. Finally, we computationally predicted the 3D structures of these conserved lncRNA-encoded peptides. To the best of our knowledge, this is the first time that hundreds of circadianly expressed zebrafish larval lncRNAs have been systematically studied. Our novel datasets, computational findings, and research framework will help circadian biologists to select circadianly expressed lncRNAs for conducting further functional investigations.

## 2. Materials and Methods

### 2.1. Fish Husbandry and Embryo Production

Wild-type AB strain zebrafish were raised at the Soochow University Zebrafish Facility according to standard protocols [40]. Wild-type embryos were produced by pair mating and then raised in E3 (5 mM NaCl, 0.17 mM KCl, 0.33 mM CaCl2, and 0.33 mM MgSO4) embryo medium at 28.5 °C. To obtain larvae under constant dark (DD) or constant light (LL) conditions, embryos were first raised under the normal light/dark (LD, 14/10 h) condition for the first four days post fertilization (dpf) to activate and entrain the circadian system, and then transferred into the DD or LL environment. The samples were collected in darkness by employing a faint red flashlight under the DD condition. The larvae were anesthetized by ice. All the samples were collected within 2 min. Total RNAs were extracted from approximately 50 of the wild-type larvae every four hours from 120–140 hpf under the constant dark or constant light conditions using TRIzol reagent (Invitrogen, Carlsbad, CA, USA). All procedures were approved by the Soochow University Animal Care and Use Committee (#SUDA20211013A01) and were in accordance with regulations of the government of the People’s Republic of China.

### 2.2. Deep Sequencing-Based Transcriptome Analysis

Total RNAs extracted from six stages, 120 hpf (CT0), 124 hpf (CT4), 128 hpf (CT8), 132 hpf (CT12), 136 hpf (CT16), and 140 hpf (CT20) were examined on 1% agarose gels for integrity. Their concentrations were examined with a Nanodrop 2000 spectrophotometer (Thermo scientific, Waltham, MA, USA). A total amount of 3 µg RNA per sample was used in the sequencing library preparations, which were generated using NEBNext^®^Ultra^TM^ RNA Library Prep Kit (Ipswich, MA, USA) following the manufacturer’s instructions. Construction and sequencing of complementary DNA (cDNA) libraries were conducted at Biomarker Technologies Corporation (Beijing, China). Clustering of the index-coded samples was performed on a cBot Cluster Generation System using TruSeq PE Cluster Kit (Illumina, PE-401-3001) according to the manufacturer’s instructions. After clustering, the library preparations were sequenced on an Illumina Hiseq 2000 platform. We used Perl scripts for removing the adapters for clean reads, calculated the Q20, Q30, and GC content, and duplication data, and then generated the raw reads. All the analysis in this study is based on clean FPKM (fragments per kilo base per million mapped reads) data with high quality.

### 2.3. Zebrafish Larval RNA-Seq Datasets under DD and LL Conditions

We generated time-course data under both DD and LL conditions from the transcriptome analysis [10] of wild-type zebrafish larvae. The two replicates were collected at six time points with a 4-h long interval. The six time point data under the constant darkness (DD) condition included WTDD120 hpf (CT0), WTDD124 hpf (CT4), WTDD128 hpf (CT8), WTDD132 hpf (CT12), WTDD136 hpf (CT16), and WTDD140 hpf (CT20), whereas the data under the constant light (LL) condition included WTLL120 hpf (CT0), WTLL124 hpf (CT4), WTLL128 hpf (CT8), WTLL132 hpf (CT12), WTLL136 hpf (CT16), and WTLL140 hpf (CT20). We compared the sequences from both replicates under the same condition, i.e., Sample 1 WT (DD) and Sample 2 WT (DD) as well as Sample 1 WT (LL) and Sample 2 WT (LL), to find the common transcripts that turned up in both samples for each condition. The sequences that matched with an E-value close to 0 were considered to be the same one in both samples in each of the DD and LL conditions. The data for each of the time points from CT0 to CT20 were determined by averaging the time-course-specific numeric expression levels from each sample. Together, we generated 3446 unannotated transcripts with six-point data under both LL and DD conditions. The expression profiles of 3446 transcripts are listed in the Supplementary Table S1 (DD) and Supplementary Table S2 (LL).

### 2.4. Identification of Zebrafish Larval lncRNAs

In order to identify the lncRNAs from the 3446 unannotated zebrafish larval transcripts, we compared these larval transcript sequences with the known zebrafish lncRNA sequences from the ZFLNC lncRNA database [29] and uncovered 3048 larval transcripts, out of the 3446 unannotated transcripts, which shared high similarities, with E-values lower than E-50, with ZFLNC lncRNAs. However, the remaining 398 larval transcripts could not be annotated with the ZFLNC database due to their higher E-values (>10^−50^). We hypothesized that the lncRNAs catalogued in the ZFLNC database were far from complete. Hence, it is possible that the E-value-based sequence comparisons may not be able to identify some of the zebrafish larval lncRNAs. Several state-of-the-art computational tools [39] can be employed to identify the coding potentials of the unannotated transcripts. A previous study revealed that the Coding Potential Assessing Tool (CPAT) [41] has sensitivity and specificity of 0.96 and 0.97, respectively, to assess the coding potential of the novel transcripts. Therefore, we analyzed the coding abilities of the remaining 398 larval transcripts with the CPAT. Out of 398 unannotated transcripts, 172 transcripts were found to have coding abilities lower than 0.01. Hence, we designated these 172 transcripts as additional lncRNAs. With the ZFLNC database-annotated 3048 lncRNAs and CPAT-determined 172 lncRNAs, we obtained a total of 3220 lncRNAs from zebrafish larvae (Supplementary Table S3). The identifiers of the lncRNAs, such as Gene Bank IDs, and Ensemble IDs, were taken from the ZFLNC database as well as NCBI BLAST search results (Supplementary Table S3).

### 2.5. Collection of Zebrafish Pineal Gland and Testis lncRNAs

To compare zebrafish larval lncRNAs with those of two different zebrafish organs/tissues, we exploited the rhythmically expressed 586 and 165 lncRNAs from the zebrafish pineal gland and testis, respectively, from our recently published study [8].

### 2.6. Identification of Zebrafish Larval lncRNAs Displaying Circadian Expression

We employed MetaCycle [42] to determine the rhythmicity of zebrafish larval lncRNAs with a statistical significance *p*-value of 0.05. Out of the 3220 zebrafish larval lncRNAs, the MetaCycle analysis determined 269 and 309 lncRNAs displaying circadian expression under DD and LL conditions, respectively (Supplementary Tables S4 and S5). For visualization of the expression patterns of these circadianly expressed lncRNAs, we further applied the BioDare2 system (https://biodare2.ed.ac.uk/, accessed on 1 October 2021) [43].

### 2.7. Investigating Circadian Regulation of Zebrafish Larval lncRNAs Mediated by E-Box, D-Box and RORE Regulatory Motifs

Numerous studies suggest the regulation of rhythmic expression of genes by *cis* regulatory motifs [8,44,45]. In particular, E-Box, D-Box and RORE motifs can regulate the expression profiles in the morning, evening, and night, respectively [46,47,48]. However, due to the lack of the promoter sequence of some lncRNAs, we investigated the promoter sequences of 115 (DD) and 112 (LL) circadianly expressed zebrafish larval ncRNAs (Supplementary Figure S1B) [49]. We BLASTed these larval lncRNAs against the NCBI’s Nucleotide collection (nt) database and selected the orthologs based on E-value-based sequence similarity and determined the Gene Bank Accession Number for each of the orthologs. The Gene Bank Accession Numbers were mapped to Ensemble IDs by bioDBnet (biological DataBase network) [50]. Supplementary Tables S6–S8 list Ensemble IDs of zebrafish larval lncRNAs under the DD condition, whereas Supplementary Tables S9–S11 show Ensemble IDs of zebrafish larval lncRNAs under the LL condition. Further, we downloaded a 5000-nucleotide 5′ upstream promoter sequence for each of the Ensemble IDs from Ensembl BioMart. We searched the E-Box, D-Box and RORE elements with a Matlab (The Mathworks, Inc., Natick MA, USA) program and the motif search tool Find Individual Motif Occurrences (FIMO) [51]. We downloaded the probability distribution for each of the E-Box, D-Box and RORE elements in FIMO from JASPAR [52] and kept the *p*-value threshold to 0.01 in the FIMO motif search. The promoter analysis helped establish a direct correlation between promoter elements and rhythmic expression of these zebrafish larval lncRNAs.

### 2.8. GO, COG, and KEGG Enrichment and Annotation of Circadianly Expressed Zebrafish Larval lncRNAs

We conducted Gene Ontology (GO) [53] analysis, Clusters of Orthologous Groups (COGs) [54] analysis, and Kyoto Encyclopedia of Genes and Genomes (KEGG) pathway analysis for all these circadianly expressed zebrafish larval lncRNAs. The GO analysis was performed with a Cytoscape open source Java application (https://cytoscape.org, accessed on 1 October 2021) [55] as well as with Blast2GO [56] software that employs the BLAST [57] algorithm to perform sequence alignment with the sequences in the nucleotide collection (nt) database in order to determine potential orthologs and the GO annotations. For all the lncRNAs orthologs with known Ensembl IDs, we also used BMKCloud (https://international.biocloud.net/, accessed on 1 October 2021) tools to visualize the GO annotation as well as to perform COGs analysis. A KEGG pathway enrichment analysis was performed with both BMKCloud and Database for Annotation, Visualization and Integrated Discovery (DAVID) v6.8 (https://david.ncifcrf.gov/, accessed on 1 October 2021) [58].

### 2.9. Principal Component Analysis (PCA) of Circadian Zebrafish Larval lncRNAs

We applied Principal Component Analysis (PCA) to compress the multidimensional time-course data into the two most important principal components [59]. The PCA assigned different scores derived from the gene expression profiles for each of the lncRNAs for all the principal components. We separately performed PCA for expression profiles of the morning, evening, and night lncRNAs. The absolute values of the PCA scores from the first principal component were used to rank the lncRNAs. The top-ranked representative lncRNAs from both the WT (DD) and WT (LL) datasets were plotted for visualization.

### 2.10. Predicting Orthologs of Zebrafish Larval lncRNAs with NCBI BLAST

We employed both local and NCBI BLAST algorithms implemented in Blast2GO [56] to compare the DNA sequences. The execution of the BLAST algorithm provided expected value (E-value) output as the measure of statistical significance of the similarity for a sequence comparison. A lower E-value (≤10^−5^) represented a match of high statistical significance.

### 2.11. Uncovering 3D Models and Functions of lncRNA-Encoded Peptides

The amino acid sequences for the selected zebrafish larval lncRNAs and their mouse and human orthologs were translated into their corresponding peptide sequences with the ExPASy translate tool [60]. We separately applied the Clustal Omega (https://www.ebi.ac.uk/Tools/msa/clustalo/, accessed on 1 October 2021) tool to perform multiple sequence alignment for each of these selected zebrafish larval lncRNA-encoded peptides with the peptides encoded by testicular and pineal gland orthologs, as well as the mouse and human orthologs. The 3D models of conserved peptides were predicted with the (PS)2-v2: Protein Structure Prediction Server [61,62] and subsequently visualized with Jmol (http://www.jmol.org/, accessed on 1 October 2021).

## 3. Results

### 3.1. Circadianly Expressed Zebrafish Larval lncRNAs under Constant Darkness (DD) Condition and Their GO, COG, and KEGG Analyses

**Figure 1:**
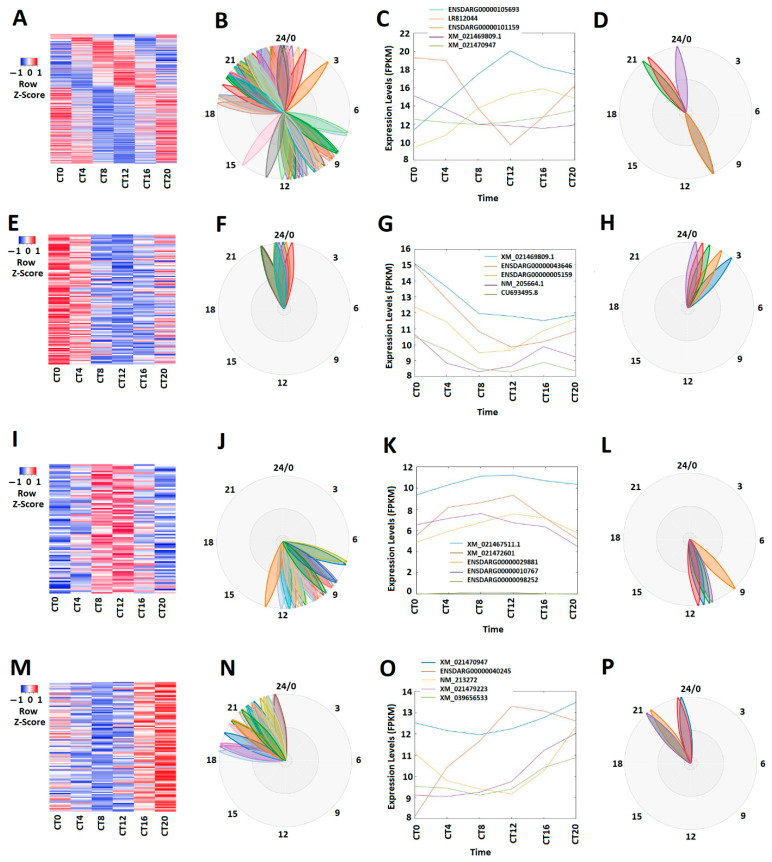
Expression profile analysis of circadianly expressed zebrafish larval lncRNAs under the DD condition. (**A**–**D**) Analysis of all the 269 circadianly expressed larval lncRNAs under the DD condition. Heat map (**A**) and BioDare2 plots (**B**) of all the 269 circadianly expressed zebrafish larval lncRNAs, expression profiles (**C**) and BioDare2 plots (**D**) of representative lncRNAs. (**E**–**H**) Analysis of 100 larval morning (CT0 and CT4) lncRNAs. Heat map (**E**) and BioDare2 plots (**F**) of the 100 larval zebrafish morning lncRNAs, expression profiles (**G**) and BioDare2 plots (**H**) of zebrafish larval morning representative lncRNAs. (**I**–**L**) Analysis of 75 zebrafish larval evening (CT8 and CT12) lncRNAs. Heat map (**I**) and BioDare2 plots (**J**) of the 75 larval evening lncRNAs, expression profiles (**K**) and BioDare2 plots (**L**) of larval evening representative lncRNAs. (**M**–**P**) Analysis of 94 larval zebrafish night (CT16 and CT20) lncRNAs. Heat map (**M**) and BioDare2 plots (**N**) of the 94 larval zebrafish night lncRNAs, expression profiles (**O**) and BioDare2 plots (**P**) of larval zebrafish night representative lncRNAs.

The circadian clock genes display unique rhythmic expression patterns at different times over the 24-h cycle. Some genes peak during the morning, some during the evening, and others at night [46,47,48]. We investigated whether these zebrafish larval lncRNAs also exhibit peak expression during morning, evening, and night. Hence, rhythmicity analysis with MetaCycle revealed that approximately 8.35% (269 out of 3220) lncRNAs displayed circadian rhythmicity under the DD condition (Figure 1A–D; Supplementary Table S4). The correlative expression profiles of these 269 circadianly expressed larval lncRNAs are shown in a heat map (Figure 1A), while their phases are exhibited as BioDare2 plots (Figure 1B). We performed PCA of these circadianly expressed larval lncRNAs and ranked them based on the scores of the first principal component (Supplementary Figure S2A). The expression patterns and phases of the top-ranked representative lncRNAs are shown in Figure 1C,D. Subsequently, based on the expression patterns, we classified these 269 larval lncRNAs into 100 morning (CT0 and CT4) lncRNAs (E-H), 75 evening (CT8 and CT12) lncRNAs (I-L), and 94 night (CT16 CT20) lncRNAs under the DD condition (Supplementary Table S5), which were ranked according to their PCA scores (Supplementary Figure S2B,D). As visualized by expression patterns (Figure 1G,K,O) and the BioDare2 plots (Figure 1H,L,P) for representative lncRNAs, distinct expression patterns were observed for these morning lncRNAs, evening lncRNAs and night lncRNAs.

We hypothesized that the circadian expression of these 269 larval lncRNAs could be mediated via E-box, D-box or RORE promoter motifs. Therefore, we searched for the presence of these promoter motifs in the 5′ promoter sequences of each of these 100 morning lncRNAs, 75 evening lncRNAs, and 94 night lncRNAs. However, due to the lack of promoter sequences of some larval lncRNAs, we could only find the Ensembl IDs and the corresponding promoter sequences for 42 morning lncRNAs (Supplementary Table S6), 33 evening lncRNAs (Supplementary Table S7), and 40 night lncRNAs (Supplementary Table S8). Subsequently, we searched for the presence of E-box, D-box or RORE elements in these promoter sequences with both the Matlab program and FIMO online application (Supplementary Table S9). Initially, we searched the presence of the motif elements using the Matlab program (Supplementary Tables S9–S12). We found that all 42 morning lncRNAs contain the E-box variable motif CANNTG, where N can be any nucleotide (Supplementary Table S10). Further, seven out of thirty-three evening lncRNAs contain the D-box variable motif TTAYGTAA (Supplementary Table S11), while seven out of forty night lncRNAs contain the RORE elements (Supplementary Table S12). We then investigated all 114 lncRNAs’ promoter sequences with the FIMO utility. Interestingly, FIMO analysis revealed that all 42 morning lncRNAs’ promoters contain E-box motif (Supplementary Table S10), all 33 evening lncRNAs’ promoters contain D-box motif (Supplementary Table S11), and all 40 night lncRNAs’ promoters contain RORE elements (Supplementary Table S12). Taken together, these analyses suggest circadian regulation of these circadianly expressed zebrafish larval lncRNAs. Although further experimental validation would be needed to confirm these findings, our computational analyses strongly suggest that under the DD condition, the rhythmicity of zebrafish larval morning lncRNAs are likely to be mediated via E-box, the rhythmicity of zebrafish larval night lncRNAs via D-box, and the rhythmicity of zebrafish larval night lncRNAs via the RORE elements.

**Figure 2:**
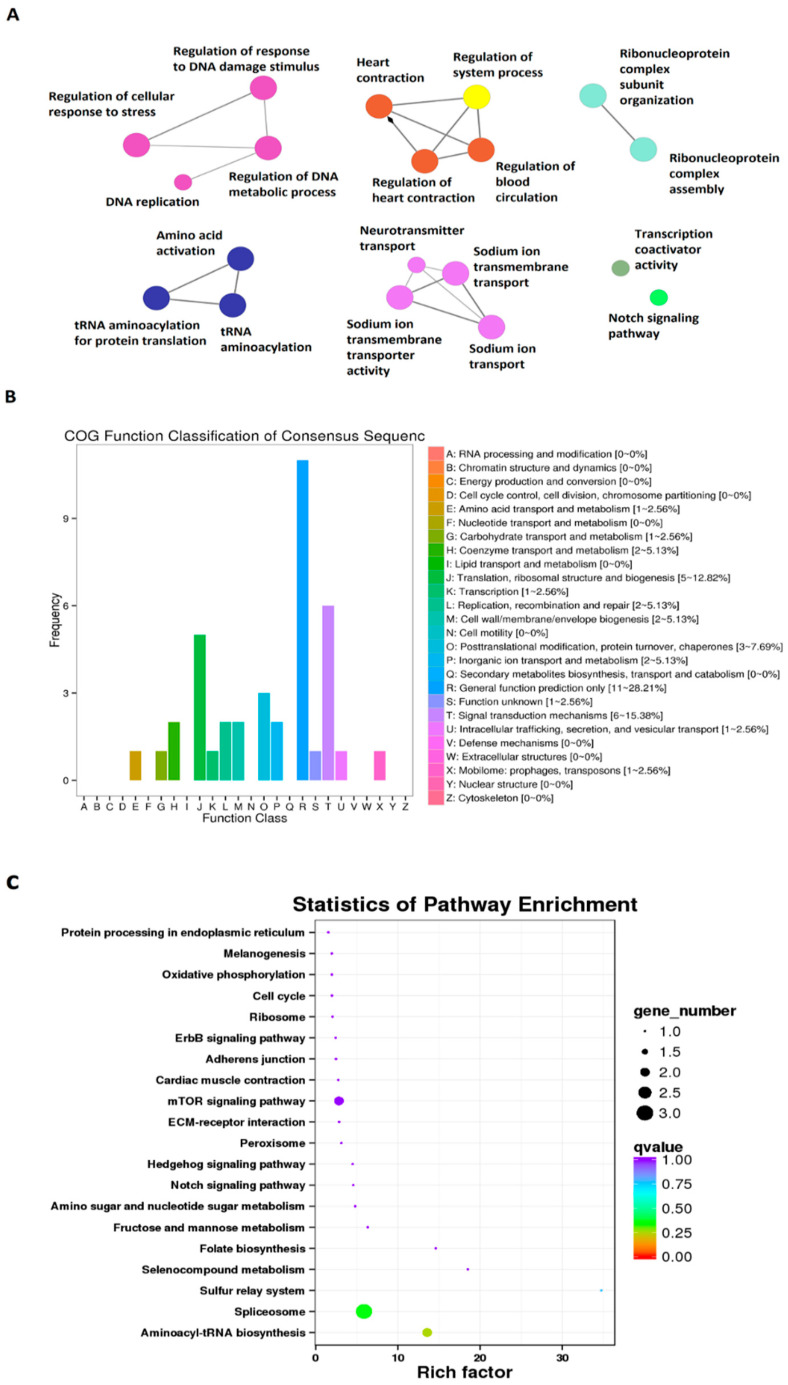
GO, COG, and KEGG analyses of circadianly expressed larval zebrafish lncRNAs under the DD condition. (**A**) GO annotation of the zebrafish larval lncRNAs under the DD condition revealed their potential involvement in several biological processes, such as nuclear receptor transcription coactivator activity, regulation of response to DNA damage stimulus, and regulation of DNA metabolic process. (**B**) COG functional classification of circadianly expressed larval zebrafish lncRNAs under the DD condition. (**C**) KEGG pathway enrichment analysis of the circadianly expressed larval zebrafish lncRNAs under the DD condition.

We then performed Gene Ontology (GO) analysis, COG functional annotation [54], and KEGG pathway enrichment analyses for all these 269 zebrafish larval lncRNAs displaying circadian rhythmicity under the DD condition (Figure 2A, Supplementary Figure S3A and Table S13). Out of the 269 lncRNAs, we were able to find the GO annotation, such as #GO, GO IDs, GO Names, and Enzyme codes, for 151 lncRNAs (Supplementary Table S13), and revealed their potential involvement in several fundamental biological activities, such as regulation of response to DNA damage stimulus, regulation of cellular response to stress, DNA replication, and regulation of DNA. The COG functional classification suggested numerous novel annotations, including amino acid transport and metabolism, translation and transcription (Figure 2B). Further, the KEGG pathway enrichment analysis uncovered gene numbers and corresponding *q*-values enriched in a diverse set of pathways, including Wnt signaling and sugar metabolism (Figure 2C, Supplementary Figure S3B and Table S14). Among the morning, evening and night lncRNAs with the known orthologs Ensemble IDs (Supplementary Tables S6–S8), we found 75 lncRNAs could be involved in different biological pathways (Supplementary Figure S3B). For instance, lncRNA Danio_rerio_newGene_6700 (FP245396.14) is potentially involved in Wnt signaling and melanogeneis, whereas lncRNAs Danio_rerio_newGene_21806 (ENSDARG00000032997) could be involved in protein processing in the endoplasmic reticulum. Taken together, we uncovered 269 zebrafish larval lncRNAs displaying circadian rhythmicity under the DD condition and determined their GO, COG and KEGG pathway enrichments.

### 3.2. Circadianly Expressed Zebrafish Larval lncRNAs under Constant Light (LL) Condition and Their GO, COG, and KEGG Analyses

**Figure 3:**
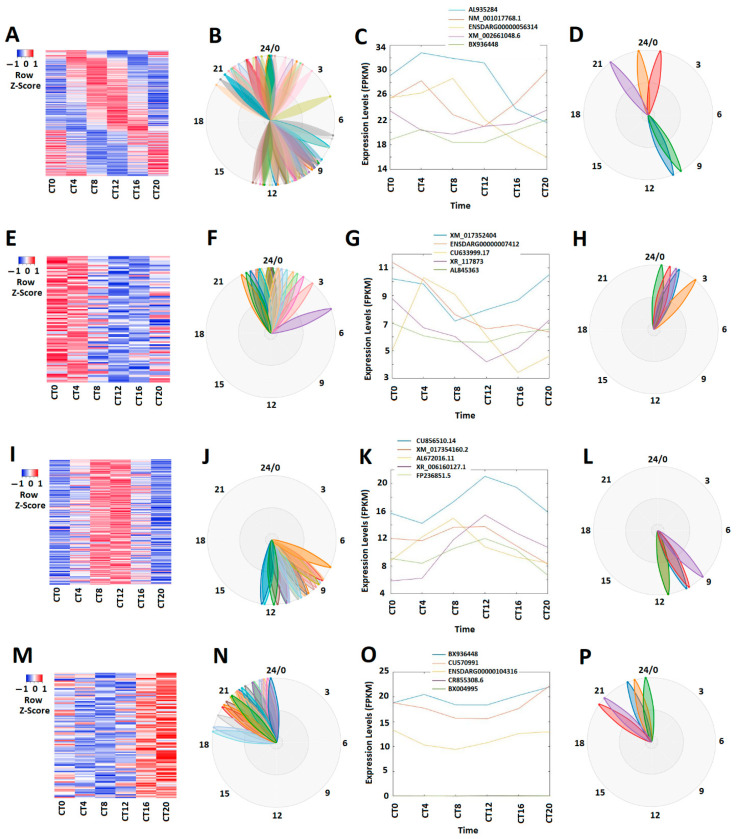
Expression profile analysis circadianly expressed zebrafish larval lncRNAs under the LL condition. (**A**–**D**) Analysis of all the 309 circadianly expressed larval lncRNAs under the DD condition. Heat map (**A**) and BioDare2 plots (**B**) of all 309 the circadianly expressed larval lncRNAs, expression profiles (**C**) and BioDare2 plots (**D**) of representative lncRNAs. (E-H) Analysis of the 78 larval morning (CT0 and CT4) lncRNAs. Heat map (**E**) and BioDare2 plots (**F**) of the 78 larval morning lncRNAs, expression profiles (**G**) and BioDare2 plots (**H**) of larval morning representative lncRNAs. (**I**–**L**) Analysis of 144 larval evening (CT8 and CT12) lncRNAs. Heat map (**I**) and BioDare2 plots (**J**) of 144 larval evening lncRNAs, expression profiles (**K**) and BioDare2 plots (**L**) of larval evening representative lncRNAs. (**M**–**P**) Analysis of 87 larval night (CT16 and CT20) lncRNAs. Heat map (**M**) and BioDare2 plots (**N**) of 87 larval night lncRNAs, expression profiles (**O**) and BioDare2 plots (**P**) of larval night representative lncRNAs.

Similar rhythmicity analysis revealed approximately 9.6% (309 out of 3220) larval lncRNAs showing circadian expression under the LL condition (Supplementary Table S15). The correlative expression profiles (Figure 3A) and expression phases (Figure 3B) of these 309 circadianly expressed larval lncRNAs under the LL condition are shown as heat map and BioDare2 plots. These 309 circadianly expressed larval lncRNAs were ranked based on their PCA scores (Supplementary Figure S4A), and the expression patterns and phases of the top-ranked representative larval lncRNAs are visualized in Figure 3C,D. We further categorized these circadianly expressed lncRNAs into 78 morning (CT0 and CT4) lncRNAs (Figure 3E–H), 144 evening (CT8 and CT12) lncRNAs (Figure 3I–L), and 87 night (CT16 and CT20) lncRNAs (Figure 3M–P) (Supplementary Table S16). For each of the morning, evening, and night lncRNAs, we separately performed PCA (Supplementary Figure S4B–D) and ranked the lncRNAs according to their PCA scores (Figure 3G,K,O). BioDare2 plots show the distinct expression patterns for these morning lncRNAs, evening lncRNAs, and night lncRNAs (Figure 3F,H,J,L,P).

Subsequently, the 5′ promoter sequences of all these circadianly expressed lncRNAs under the LL condition were investigated (Supplementary Tables S17–S23). Out of the 78 morning lncRNAs, 144 evening lncRNAs, and 87 night lncRNAs, we were able to find the Ensembl IDs and the corresponding promoter sequences of 30 morning lncRNAs (Supplementary Table S17), 58 evening lncRNAs (Supplementary Table S18), and 24 night lncRNAs (Supplementary Table S19). Together, we found 112 zebrafish larval lncRNAs under the LL condition with known Ensemble IDs. Similarly, we initially searched for the presence of the E-box, D-box or RORE promoter motifs in the 5′ promoter regions of these larval lncRNAs. The batch search analysis of motif sequences with the Matlab program revealed that all 30 morning lncRNAs’ promoter regions contain the E-box variable motifs (Supplementary Tables S20 and S21), whereas 14 evening lncRNAs’ promoter regions contain the D-box (Supplementary Table S22) and only one night lncRNAs’ promoter region contains RORE motifs (Supplementary Table S23). We further interrogated these promoter regions with the FIMO utility. Interestingly, FIMO analysis showed that all 30 morning lncRNAs’ promoter regions contain the E-box motifs (Supplementary Tables S20 and S21), all 58 evening lncRNAs’ promoter regions contain D-box (Supplementary Table S22), and all 24 night lncRNAs’ promoter regions contain RORE motifs (Supplementary Table S23). Our findings, despite constrained by the availability of the promoter region sequences of lncRNAs, strongly suggest circadian regulation of these circadianly expressed zebrafish larval lncRNAs under LL condition. In particular, the zebrafish larval morning lncRNAs, evening lncRNAs, and night lncRNAs are likely to be mediated via the E-box, D-box or RORE promoter motifs, respectively.

**Figure 4:**
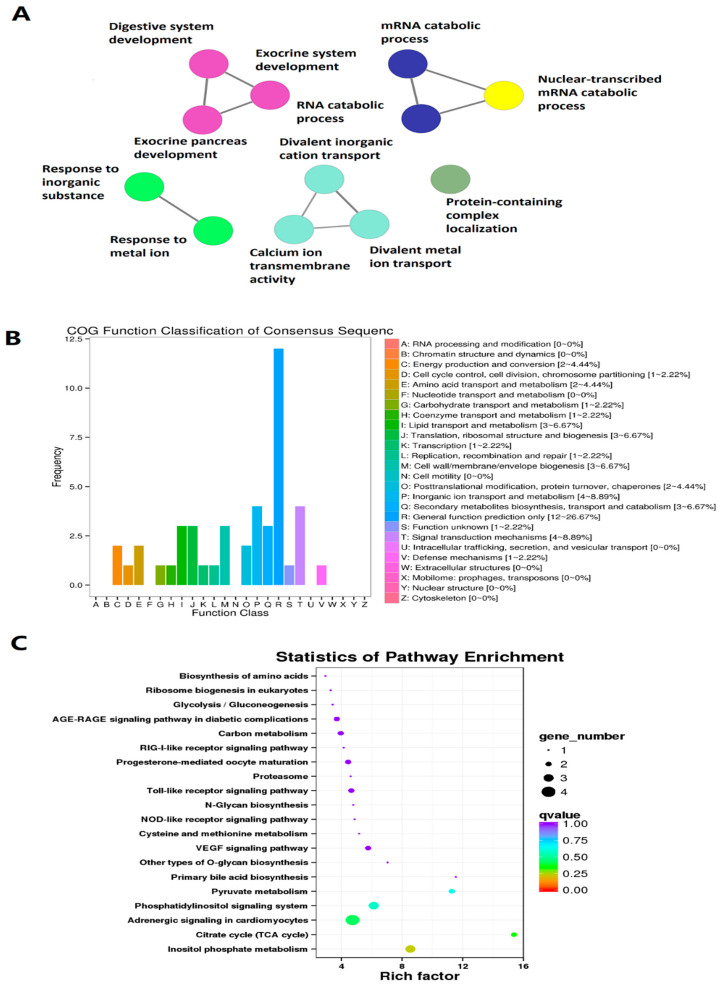
GO, COG, and KEGG analyses of circadianly expressed zebrafish larval lncRNAs under the DD condition. (**A**) GO annotation revealed the lncRNAs potentially involved in different biological processes, such as nuclear-transcribed mRNA catabolic process, and eye photoreceptor cell differentiation. (**B**) COG functional classification of circadianly expressed larval lncRNAs under the DD condition. (**C**), KEGG pathway enrichment analysis of circadianly expressed lncRNAs under the DD condition.

We also performed GO, COG, and KEGG pathway analyses of these zebrafish larval lncRNAs under the LL condition with known orthologs. Out of the 309 circadianly expressed lncRNAs under the LL condition, we were able to determine GO annotations for 173 lncRNAs with various genomic annotations, such as GO IDs, GO Names (Supplementary Table S24). In particular, GO analysis established the potential roles of circadianly expressed lncRNAs in a diverse set of fundamental biological processes, such as digestive system development, exocrine system development, RNA catabolic process, exocrine pancreas development, mRNA catabolic process, nuclear-transcribed mRNA catabolic process, response to inorganic substances, and response to metal ion protein-containing complex localization (Figure 4A, Supplementary Figure S5A and Table S24). The COG classification elucidated potential involvement of these zebrafish lncRNAs in important biological activities, such as cell cycle control, cell division, translation, transcription, and amino acid transport and metabolism (Figure 4B). KEGG pathway enrichment analysis determined statistics of pathway enrichment as well as direct role of lncRNAs in biological activities. For example, the KEGG statistics of pathway enrichment highlighted gene numbers with statistically significant *q*-values that regulated cellular activities and pathways (Figure 4C, Supplementary Figure S5B and Table S25). Further, 93 zebrafish larval lncRNAs under the LL condition are potentially involved in multiple biological pathways. For example, a zebrafish larval lncRNA Danio_rerio_newGene_5943 (ENSDARG00000104170) is potentially involved in nucleic acid binding and metal ion binding, the other lncRNA, Danio_rerio_newGene_17037 (ENSDARG00000093359), is likely to be involved in inositol phosphate metabolism, metabolic pathways, and the phosphatidylinositol signaling system (Supplementary Table S25). The percentages of genes potentially involved in biological processes, cellular components, and molecular functions are shown in Supplementary Figure S5B. Overall, we determined 309 circadianly expressed larval lncRNAs under the LL condition and their GO, COG, and KEGG pathway annotation analyses suggest these circadianly expressed lncRNAs likely contribute to various biological processes.

### 3.3. Circadianly Expressed Zebrafish Larval lncRNAs Are Coexpressed under Both Constant Darkness (DD) and Constant Light (LL) Condition and Their GO, COG, and KEGG Analyses

**Figure 5:**
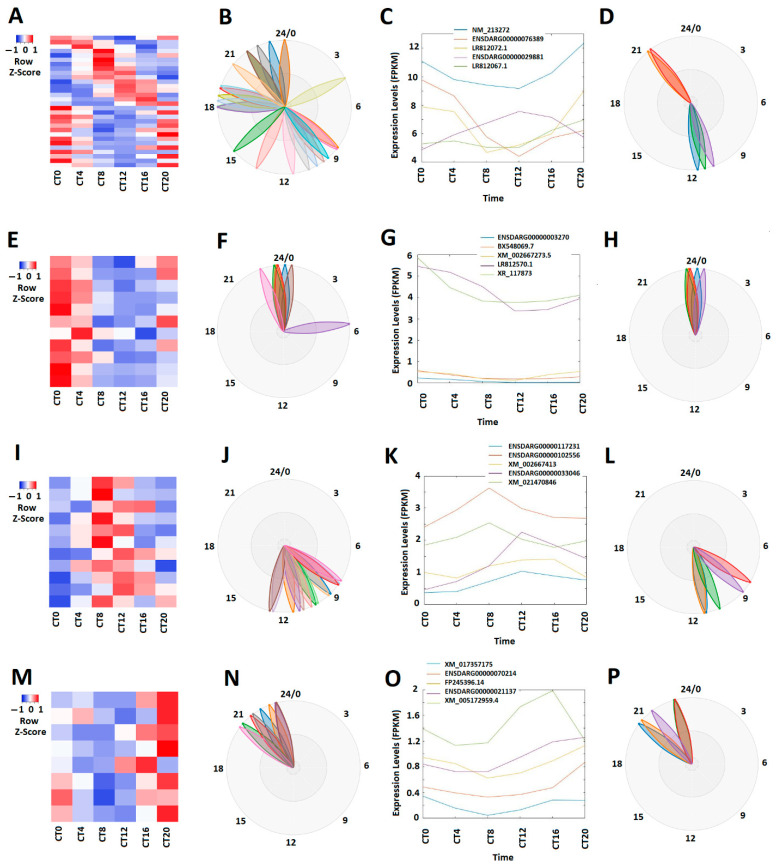
Expression profile analysis of circadianly expressed zebrafish larval lncRNAs coexpressed under both the DD condition and the LL condition. (**A**–**D**) Analysis of all the 30 coexpressing larval lncRNAs with expression profiles from DD condition. Heat map (**A**) and BioDare2 plots (**B**) of all the 30 coexpressing larval lncRNAs, expression profiles (**C**) and BioDare2 plots (**D**) of representative lncRNAs. (**E**–**H**) Analysis of the 11 coexpressing larval morning (CT0 and CT4) lncRNAs. Heat map (**E**) and BioDare2 plots (**F**) of the 11 larval morning lncRNAs, expression profiles (**G**) and BioDare2 plots (**H**) of larval morning representative lncRNAs. (**I**–**L**) Analysis of the eight coexpressing larval evening (CT8 and CT12) lncRNAs. Heat map (**I**) and BioDare2 plots (**J**) of eight larval evening lncRNAs, expression profiles (**K**) and BioDare2 plots (**L**) of larval evening representative lncRNAs. (**M**–**P**) Analysis of eight coexpressing larval night (CT16 and CT20) lncRNAs. Heat map (**M**) and BioDare2 plots (**N**) of eight larval night lncRNAs, expression profiles (**O**) and BioDare2 plots (**P**) of larval night representative lncRNAs.

In order to understand the effect of light on gain or loss of rhythmicity we compared circadianly expressed lncRNAs under the DD condition with those under LL condition and found that a total of 239 lncRNAs that were circadianly expressed under the DD condition, but not the LL condition (Supplementary Table S26, Figure 5 and Supplementary Figure S6), implicating loss of rhythmicity, likely due to exposure to constant light. Interestingly, a total of 279 lncRNAs were circadianly expressed only under the LL condition (Supplementary Table S26 and Figures S7 and S8), implying gain of rhythmicity under constant light. Further, 30 lncRNAs were coexpressed under both DD and LL conditions (Supplementary Table S26). Heat map and BioDare2 plots show the correlative expression profiles (Figure 5A) and phases (Figure 5B) of these 30 coexpressing lncRNAs. The expression patterns and phases of the top-ranked lncRNAs based upon their PCA scores (Supplementary Figure S6A) are shown in Figure 5C,D. We further categorized these 30 lncRNAs into morning, evening, and night lncRNAs.

Based on the expression profiles under the DD condition, we found eleven, eleven and eight lncRNAs exhibited peak expression in the morning (Figure 5E–H), evening (Figure 5I–L), and night (Figure 5M–P), respectively (Supplementary Table S27). Further, we separately performed PCA of the expression profiles of these morning lncRNAs, evening lncRNAs, and night lncRNAs under the DD condition (Supplementary Figure S6B–D). BioDare2 plots show the distinct expression patterns for the morning lncRNAs, evening lncRNAs, and night lncRNAs (Figure 5F,H,J,H,N,P). In contrast, based on the expression profiles of 30 lncRNAs under LL conditions, there were nine, fourteen and seven lncRNAs that peaked in the morning (Supplementary Figure S7E–H), evening (Supplementary Figure S7I–L), and night (Supplementary Figure S7M–P), respectively (Supplementary Table S28). PCA of the expression profiles of these morning lncRNAs, evening lncRNAs, and night lncRNAs under the LL condition allowed for selecting top-ranked representatives (Supplementary Figure S8A–D). BioDare2 plots show the distinct expression patterns for the morning lncRNAs, evening lncRNAs, and night lncRNAs (Supplementary Figure S7F,H,J,N,P). Similarly, promoter analysis suggested that rhythmicity of these 30 circadianly expressed zebrafish larval lncRNAs under both DD (Supplementary Tables S9–S12) and LL (Supplementary Tables S20–S23) conditions are likely mediated via the E-box, D-box or RORE motifs in the morning, evening, and night, respectively.

We also compared the changes in BioDare2 phase values of the 30 lncRNAs under DD and LL conditions (Supplementary Table S28). The results suggest that 12 lncRNAs gained phase due to exposure to light as their phase values increased from the DD to the LL condition, while 17 lncRNAs exhibited decreased phase values under the LL condition. Interestingly, one lncRNA Danio_rerio_newGene_3412 (ENSDARG00000029881) showed exactly the same phase value under both the DD and LL condition because of very similar expression values under both DD and LL conditions (Supplementary Table S28).

**Figure 6:**
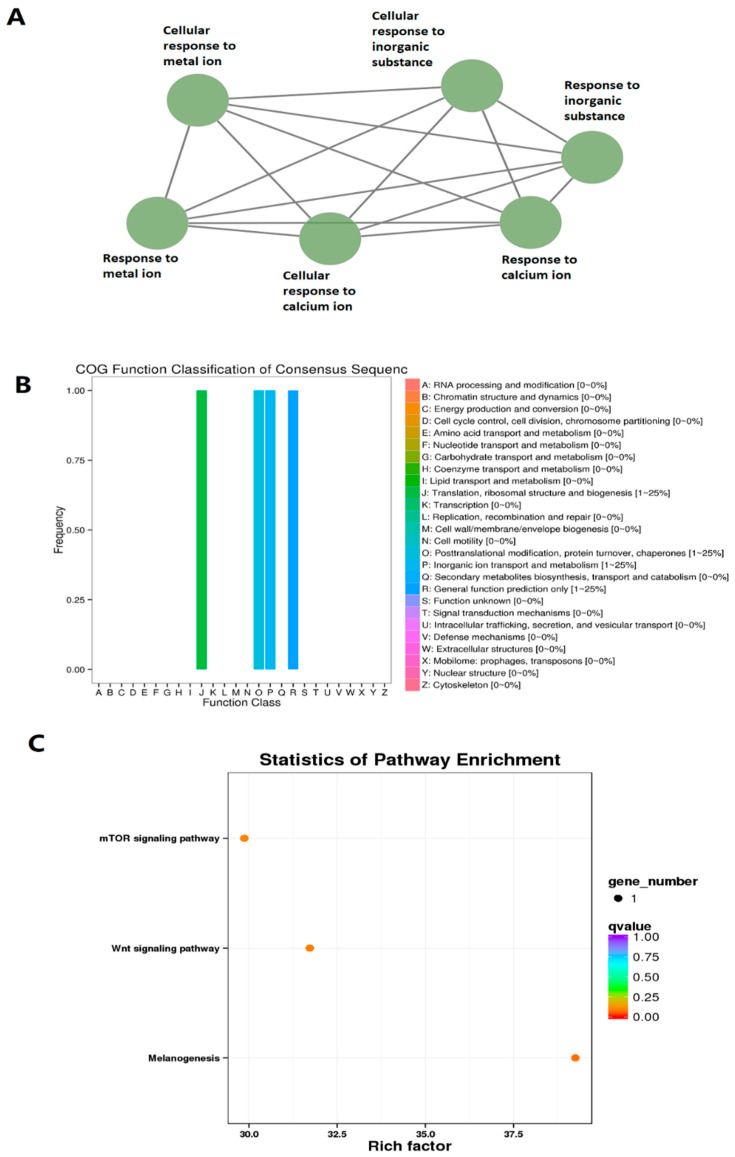
GO, COG, and KEGG analyses of circadianly expressed zebrafish larval lncRNAs coexpressed under both DD and LL conditions. (**A**) GO annotation revealed the lncRNAs potentially involved in different biological processes, such as cellular protein modification process, protein modification process, cellular macromolecule metabolic process, and protein metabolic process. (**B**) COG functional classification of circadianly expressed zebrafish larval lncRNAs coexpressed under both DD and LL conditions. (**C**) KEGG pathway enrichment analysis of circadianly expressed zebrafish larval lncRNAs coexpressed under both DD and LL condition.

All 30 coexpressed lncRNAs were investigated to determine their GO, COG, and KEGG pathway annotations. GO analysis suggested the potential roles of these coexpressed circadian lncRNAs in diverse sets of biological processes, such as cellular response to metal ions, cellular response to calcium ions, and cellular response to inorganic substances (Figure 6A, Supplementary Figure S9A and Table S13). The COG classification elucidated their involvement in important biological activities, such as posttranslational modification, protein turnover, and chaperones (Figure 6B). The KEGG pathway analysis determined the statistics of pathway enrichment as well as the possible roles of lncRNAs in biological activities (Figure 6C, Supplementary Figure S9B and Table S25). Numerous circadianly expressed zebrafish larval lncRNAs are potentially involved in multiple biological pathways. For example, a zebrafish larval lncRNA Danio_rerio_newGene_11483 (ENSDARG00000098252) is likely to be involved in calcium-dependent membrane targeting, whereas the other lncRNA Danio_rerio_newGene_6700 (ENSDARG00000063437) is likely to be involved in Wnt signaling and melanogenesis. Taken together, we identified 30 zebrafish larval lncRNAs coexpressed under both DD and LL conditions and predicted their possible functional roles.

### 3.4. Circadianly Expressed Zebrafish Larval lncRNAs Are Coexpressed in the Pineal Gland and the Testis

**Figure 7:**
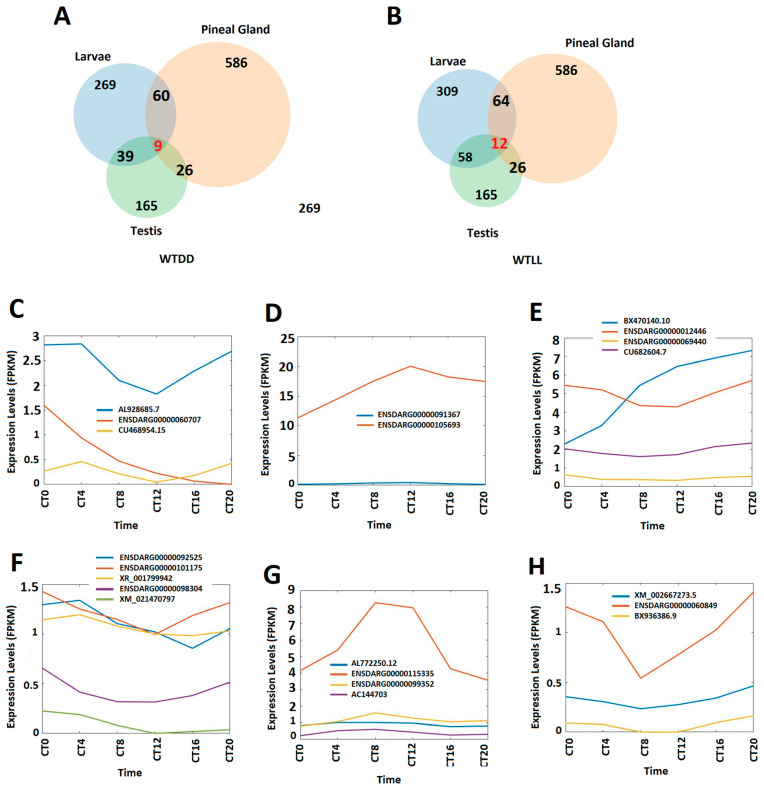
Circadianly expressed zebrafish larval lncRNAs are coexpressed in the zebrafish pineal gland and testis. (**A**) Circadianly expressed lncRNAs coexpressed in larvae, pineal gland and testis under DD condition. (**B**) Circadianly expressed lncRNAs coexpressed in larvae, pineal gland and testis under LL condition. (**C**–**E**) Expression profiles of representative lncRNAs under DD condition: three zebrafish larval morning (CT0 and CT 4) lncRNAs (**C**), two zebrafish larval evening (CT8 and CT12) lncRNAs (**D**), and four zebrafish larval night (CT16 and CT20) lncRNAs (**E**). (**F**–**H**) Expression profiles of representative lncRNAs under LL condition: five zebrafish larval morning lncRNAs (**F**), four zebrafish larval evening lncRNAs (**G**), and three zebrafish larval night lncRNAs (**H**).

Our recent study [8] identified 586 and 165 rhythmically expressed lncRNAs in zebrafish pineal gland and zebrafish testis, respectively, and demonstrated that 26 rhythmically expressed lncRNAs are coexpressed in both the zebrafish pineal gland and testis [8]. We hypothesized that a certain number of circadianly expressed larval lncRNAs may be rhythmically/circadianly expressed in zebrafish pineal gland and testis, even though larvae and adult organs exhibit distinct developmental programs [63,64]. Therefore, we compared these circadianly expressed zebrafish larval lncRNAs under DD and LL conditions with those of the pineal gland testis lncRNAs (Figure 7, Supplementary Tables S29–S34) and revealed that under the DD condition, 60 circadianly expressed larval lncRNAs were coexpressed in the pineal gland (Supplementary Table S29), while 39 circadianly expressed larval lncRNAs were coexpressed in the testis (Supplementary Table S30); and nine circadianly expressed larval lncRNAs were coexpressed in both the pineal gland and testis (Figure 7A, Supplementary Table S31). In contrast, under the LL condition, 64 circadianly expressed larval lncRNAs were coexpressed in the pineal gland (Supplementary Table S32), while 58 circadianly expressed larval lncRNAs were coexpressed in the testis (Supplementary Table S33); and 12 circadianly expressed larval lncRNAs under the LL condition were coexpressed in both the pineal gland and testis (Figure 7B, Supplementary Table S34).

We further analyzed the expression profiles of the circadianly expressed zebrafish larval lncRNAs from both DD and LL conditions that were coexpressed in the pineal gland and testis. Under the DD condition, we found that out of the nine lncRNAs coexpressed in the larvae, pineal gland, and testis, three lncRNAs, two lncRNAs and four lncRNAs peaked in the morning, evening and night, respectively (Figure 7A,C–E, Supplementary Table S31), while under the LL condition, out of the 12 coexpressed lncRNAs, we identified five lncRNAs, four lncRNAs and three lncRNAs peaking in the morning, evening and night, respectively (Figure 7B,F–H, Supplementary Table S34). Overall, we found that zebrafish larvae coexpress nine and twelve lncRNAs in both pineal gland and testis under DD and LL conditions, respectively, and we analyzed their expression profiles in the morning, evening and night.

### 3.5. Uncovering Coexpressing lncRNA-Encoded Peptides and Their 3D Structures

We also investigated that how many coexpressing zebrafish larval lncRNAs encode peptides. In particular, we computationally predicted the 3D structures and functions of these lncRNAs coexpressing under both DD and LL conditions. Under the DD condition, 267 peptides were computed to be encoded by these nine lncRNAs coexpressed in zebrafish larvae, pineal gland, and testis (Supplementary Table S35). In contrast, under LL condition, 226 peptides were predicted to be encoded by these 12 lncRNAs coexpressed in zebrafish larvae, pineal gland, and testis, respectively (Supplementary Table S36).

**Figure 8:**
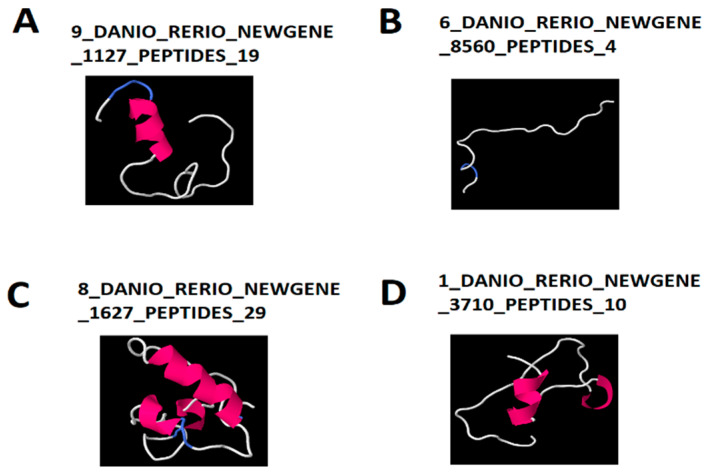
Three-dimensional structures predicted by the (PS)2-v2 Protein Structure Prediction Server for the lncRNA-encoded peptides simultaneously observed in zebrafish larvae, pineal gland and testis. Zebrafish lncRNA-encoded peptides coexpressed in larvae (DD condition), pineal gland, and testis (**A**–**C**), and Zebrafish lncRNA-encoded peptide coexpressed in larvae (LL condition), pineal gland, and testis (**D**). The 3D structures of the peptides depict the presence of α-helix (pink or purple motif structure), β-strand (yellow layered band), and random coils (white or blue thread) among the coexpressing lncRNA- encoded peptides with the known domains from Protein Data Bank such as, A (1vg5A), B (1gxrA), C (2iftA), and D (1kpfA).

We further computationally predicted the 3D structures and functions of these coexpressing lncRNA-encoded peptides (Figure 8, Supplementary Tables S37 and S38). Out of the 267 peptides encoded by the nine coexpressing larval lncRNAs under the DD condition, we were able to predict 3D structures for three lncRNA-encoded peptides including 9_DANIO_RERIO_NEWGENE_1127_PEPTIDES_19 (CU682604.7), 6_DANIO_RERIO_NEWGENE_8560_PEPTIDES_3 (ENSDARG00000105693), and 8_DANIO_RERIO_NEWGENE_1627_PEPTIDES_2 (ENSDARG00000069440) (Figure 8A–C and Supplementary Table S37). In contrast, out of the 226 peptides encoded by the 12 coexpressing larval lncRNAs under the LL condition, we were able to predict the 3D structure for one lncRNA-encoded peptide 1_DANIO_RERIO_NEWGENE_3710_PEPTIDES_10 (XM_002667273.5) (Figure 8D). The 3D structures revealed the presence of α-helix, β-strand, and random coils (Figure 8A–D) among the coexpressing lncRNA-encoded peptides. Subsequently, we analyzed the 3D structures by investigating the known domains from Protein Data Bank (http://www.rcsb.org/, accessed on 1 October 2021) to determine the conserved protein domains for each of the 3D model (Supplementary Tables S37 and S38).

Interestingly, all the predicted 3D structures mapped to the known domains in the Protein Data Bank. For example, under DD condition, the peptides 9_DANIO_RERIO_NEWGENE_1127_PEPTIDES_19(CU682604.7), and 6_DANIO_RERIO_NEWGENE_8560_PEPTIDES_3 (ENSDARG00000105693) were mapped to the known domains 1vg5A and 1gxrA, respectively (Figure 8). As another example, under the condition, the peptide 1_DANIO_RERIO_NEWGENE_3710_PEPTIDES_10 (XM_002667273.5) was mapped to the known domains 1kpfA. Together, we predicted 3D structures for the four lncRNA-encoded peptides and determined their domains from the Protein Data Bank.

### 3.6. Conservation of Circadianly Expressed Zebrafish Larval lncRNAs under DD and LL Conditions with Mice and Humans

**Figure 9:**
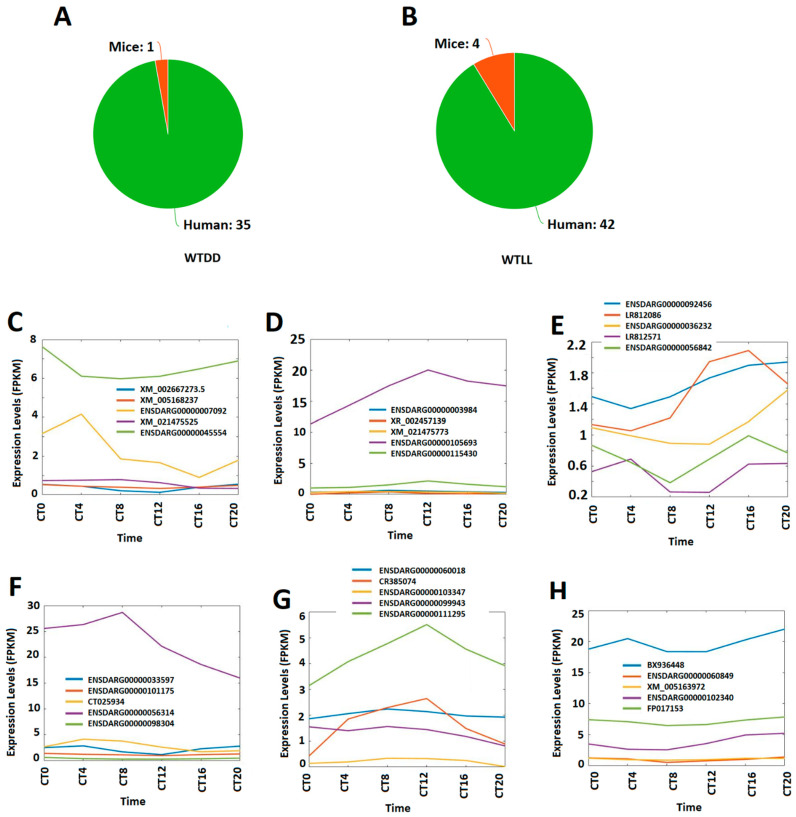
Conservation of circadianly expressed larval lncRNAs in humans and mice, and classification of lncRNAs conserved with humans into the morning, evening, and night groups. The numbers of human and mouse orthologs of the circadianly expressed zebrafish larval lncRNAs under DD condition (**A**) and LL condition (**B**). (**C**–**E**) Expression profiles of the representative larval morning (CT0 and CT4) lncRNAs (**C**), evening (CT8 and CT12) lncRNAs (**D**), and night (CT16 and CT20) lncRNAs (**E**), under the DD condition, shared with humans. (**F**–**H**) Expression profiles of the representative larval morning (CT0 and CT4) lncRNAs (**F**), evening (CT 8 and CT12) lncRNAs (**G**), and night (CT16 and CT20) lncRNAs (**H**), under the LL condition, shared with humans.

We investigated the conservation of circadianly expressed zebrafish larval lncRNAs with humans, mice, and fruit flies. In particular, we determined if some of these circadianly expressed zebrafish larval lncRNAs under DD and LL conditions, which are also coexpressed in the pineal gland and testis lncRNAs, are conserved with the aforementioned three species. In order to find the conservation of these lncRNAs, we determined human, mouse and fruit fly orthologs of these larval lncRNAs from both DD and LL conditions (Figure 9A,B, Supplementary Tables S39–S41). With the E-value-based similarity of sequences, we found that under the DD condition, 35 circadianly expressed zebrafish larval lncRNAs are conserved with humans (Supplementary Table S39), while only one circadianly expressed zebrafish larval lncRNA is conserved in mice (Supplementary Table S40), and only one circadianly expressed zebrafish larval lncRNA is conserved simultaneously in both humans and mice (Supplementary Table S41). Specifically, the larval lncRNA Danio_rerio_newGene_17148 (XM_039656533) has a human ortholog (AV748161.1) and a mouse ortholog (CF745161.1). We further divided the 35 larval lncRNAs conserved with humans into the morning, evening and night groups, and visualized the corresponding representative lncRNAs (Supplementary Table S39, Figure 9C–E). There were 12 lncRNAs, 9 lncRNAs, and 14 lncRNAs peaking in the morning, evening and night, respectively. Interestingly, out of the nine lncRNAs coexpressed in zebrafish larvae, pineal gland and testis, two lncRNAs, DANIO_RERIO_NEWGENE_18433 (ENSDARG00000012446) and DANIO_RERIO_NEWGENE_19201 (ENSDARG00000105693), are part of the 35 lncRNAs conserved with humans. However, the one larval lncRNA under DD condition conserved with mice does not belong to the nine coexpressing lncRNAs.

In contrast, under the LL condition, 42 circadianly expressed zebrafish larval lncRNAs are conserved with humans (Supplementary Table S42), while four circadianly expressed zebrafish larval lncRNAs are conserved with mice (Supplementary Table S43); and four circadian zebrafish larval lncRNAs are conserved simultaneously with both humans and mice (Supplementary Table S44). We also categorized 42 larval lncRNAs (LL) conserved with humans into the morning, evening and night groups and visualized the corresponding representative lncRNAs (Supplementary Table S42, Figure 9F–H). There were 8 lncRNAs, 26 lncRNAs, and 8 lncRNAs peaking in the morning, evening and night, respectively. Out of the 42 lncRNAs conserved with humans, three lncRNAs, DANIO_RERIO_NEWGENE_11679 (ENSDARG00000060849), DANIO_RERIO_NEWGENE_15482 (ENSDARG00000101175), and DANIO_RERIO_NEWGENE_2130 (ENSDARG00000098304), belong to the 12 lncRNAs coexpressed in the zebrafish larvae, pineal gland, and testis. However, none of the four lncRNAs conserved with mice belongs to the 12 coexpressing lncRNAs. Further, none of the circadianly expressed larval lncRNAs from either the DD or LL conditions is conserved with any in fruit flies. Taken together, our conservative analysis revealed that a higher number of zebrafish larval lncRNAs under both DD and LL conditions are more conserved with humans than with mice.

Subsequently, we analyzed the conserved lncRNA-encoded peptides from both DD and LL conditions and investigated their functions from the Protein Data Bank. Specifically, under DD condition, we identified 19, 8, and 4 peptides encoded by these conserved zebrafish larval lncRNAs, their human orthologs, and mouse orthologs, respectively (Supplementary Tables S44–S50). The conservative analysis of the lncRNA-encoded peptides revealed one peptide conserved among zebrafish larvae, mice, and humans (Supplementary Table S47). In contrast, under LL condition, we identified 132, 12, and 25 peptides encoded by the conserved zebrafish larval lncRNAs, their human orthologs, and mouse orthologs, respectively (Supplementary Tables S48–S50). However, the conservative analysis of the lncRNA-encoded peptides revealed that no peptides are conserved among zebrafish larvae, mice, and humans. Together, we uncovered one lncRNA-encoded peptide conserved among these three species.

We then performed multiple sequence alignments of the peptides encoded by this lncRNA and its orthologs to investigate its 3D structures and functions. However, there were no matching domain and 3D structures of the conserved peptides encoded by the conserved lncRNA. Overall, we uncovered one conserved peptide encoded by the lncRNA conserved among zebrafish larvae, mice, and humans.

## 4. Discussion

lncRNAs have been implicated in numerous biological processes [24,65]. Although previous studies have uncovered coding potentials [39], expression profiles [26,27,66], and numerous rhythmically expressed lncRNAs from different zebrafish organs [8], our understanding of the involvement of lncRNAs in circadian regulation remains far from complete. Despite a few studies [17,67] investigating the circadian regulation of lncRNAs, the effect of light on rhythmically expressed zebrafish larval lncRNAs has not been studied. Although our previous study [8] identified 26 rhythmically expressed lncRNAs coexpressed in zebrafish pineal gland and testis, further research was needed to investigate how many of these 26 lncRNAs are rhythmically/circadianly expressed in zebrafish larvae.

In this study, we generated time-course transcriptome profiles of zebrafish larvae (Supplementary Tables S1 and S2), employing the state-of-the-art bioinformatic tools to investigate circadianly expressed lncRNAs under both DD and LL conditions and uncovered circadian dynamics regulating the expression profiles of the zebrafish larval lncRNAs. In comparison to a recent study [19] that investigated circadian regulation of over one hundred lncRNAs in the rat pineal gland, including elucidation of the operating mechanism of circadian clocks of eight lncRNAs in the suprachiasmatic nucleus (SCN), our study identified thousands of zebrafish larval transcripts under both DD and LL conditions. Specifically, we investigated the expression profiles of 3220 lncRNAs (Supplementary Table S4) under DD and LL conditions, identified 578 circadianly expressed lncRNAs, and annotated them with GO, COG, and KEGG pathway enrichment analyses (Supplementary Tables S13, S14, S24 and S25). The computational findings suggest that most of these circadianly expressed larval lncRNAs potentially contribute to crucial biological functions.

We compared the circadianly expressed larval lncRNAs with lncRNAs from the pineal gland and testis [8], and found that zebrafish larvae coexpress nine circadianly expressed lncRNAs in both the pineal gland and testis under the DD condition (Supplementary Table S13), whereas zebrafish larvae coexpress 12 circadianly expressed lncRNAs with in both the pineal gland and testis under the LL condition (Supplementary Tables S31–S34), which belong to the 26 lncRNAs coexpressed in zebrafish pineal gland and testis we previously reported [8] (Figure 7A,B, Supplementary Tables S31 and S34). We investigated peptides encoded by these coexpressing lncRNAs to predict their 3D models and functions. In addition, we performed a conservative analysis of the larval lncRNAs with humans, mice, and fruit flies (Supplementary Tables S39–S50). We found that zebrafish larvae share as many as 35 and 42 lncRNAs with humans under DD and LL conditions, respectively, while zebrafish larvae share as many as one and four lncRNAs with mice under DD and LL conditions, respectively. Hence, we selected the five circadianly expressed lncRNAs shared by these three species, investigated the corresponding lncRNA-encoded peptides, and revealed hundreds of peptides encoded by these 5 lncRNAs. We selected these conserved peptides and investigated their 3D models and corresponding known domains from the Protein Data Bank and uncovered several peptides sharing close resemblance in terms of α-helix, β-strand, and random coils.

Although our framework, which combines novel experimental data with computational analysis, brings unprecedented insights into the circadianly expressed lncRNAs in zebrafish larvae, the study is constrained by a few limitations inherent in the bioinformatic analysis. For example, some of the peptides predicted in this study are more than 100 amino acids long. Hence, additional studies are required to investigate lncRNA-encoded micropeptides that usually contain less than 100 amino acids [31]. However, our approach, which combines biological data and computational techniques, can be applied to investigate both micropeptides and canonical peptides. Second, this study employs RNA-seq technology to investigate the lncRNAs. However, the RNA-seq technology has its own set of shortcomings [68] and often fails to identify certain lncRNAs due to the constraints imposed by poly(A) tails [69]. Third, although our comparative and conservative analysis reveals numerous interesting coexpressing/conserved lncRNAs, the numbers of such lncRNAs are far from complete. It is likely that there are more larval lncRNAs coexpressed in different organs/tissues of zebrafish or conserved with other species. However, due to the lack of experimental data and sequencing information of other tissues, finding a larger number of coexpressing/conserved lncRNAs remains an open research direction. Fourth, the FIMO tool only allows for a few specific *p*-values to detect the E-Box, D-Box and RORE elements, which may cause multiple false positives. As such, the regulation of circadianly expressed lncRNA by the E-Box, D-Box and RORE requires confirmation by wet-lab experiments. In fact, all the computational predictions require additional biological experimental validation. Fifth, although a zebrafish larva embodies a whole zebrafish, it might not be developed sufficiently to provide the best possible lncRNA expression profiles. A larva needs to undergo a long developmental process before developing an organ such as a testis, and the larval pineal gland and the adult pineal gland may use different sets of lncRNAs. It is possible that some of the lncRNAs expressed in a zebrafish larva may not be expressed in either the adult pineal gland or adult testis. Hence, comparative analysis of zebrafish larval lncRNAs with those in the pineal gland and testis requires additional experimental validations. Sixth, for several larval lncRNAs identified by similarity with ZFLNC lncRNAs (Supplementary Table S3), additional research is required to map them to the correct known identifiers in the Gene Bank or Ensembl, as the ZFLNC database lacks identifiers for thousands of lncRNAs. Finally, the effect of light on lncRNAs also requires further investigation. Despite all the limitations, our study uncovers interesting patterns derived from real experimental data. In particular, we predicted 3D models and functions of the conserved peptides encoded by the coexpressing/conserved lncRNAs. To the best of our knowledge, this is for the first time that hundreds of circadianly expressed lncRNAs have been revealed in zebrafish larvae. Our integrative framework, which combines data and bioinformatics analysis, can be expanded to investigate the circadian regulation of a diverse set of noncoding RNAs, and should help circadian biologists to select lncRNAs of interest prior to conducting time-consuming wet-lab experiments.

## 5. Conclusions

This study investigated thousands of zebrafish larval lncRNAs under DD and LL conditions, uncovered 578 circadianly expressed lncRNAs, and further determined their GO, COG, and KEGG functions. Thirty circadianly expressed larval lncRNAs are coexpressed under both DD and LL conditions. Under the DD condition, nine circadianly expressed larval lncRNAs can be found in both the pineal gland and testis, whereas under the LL condition, 12 circadianly expressed larval lncRNAs can be found in both the pineal gland and testis. The conservative analysis revealed that under the DD condition, 35 and one larval lncRNA are conserved in humans and mice, respectively, while under the LL condition, 42 and four larval lncRNAs are conserved in humans and mice, respectively. We also computationally predicted 3D models of the peptides encoded by coexpressing or conserved circadianly expressed larval lncRNAs and examined the corresponding domains in the Protein Data Bank. By integrating novel data and state-of-the-art bioinformatic methods, our study for the first time uncovers circadian regulation of zebrafish larval lncRNAs under both LL and DD conditions.

## Data Availability

The data supporting the reported results can be found in the supplementary materials.

## Supplementary Materials

The following are available online at https://www.mdpi.com/article/10.3390/cells10113173/s1. Supplementary Figure S1. Research pipeline for investigating wild-type zebrafish larval lncRNAs under DD and LL conditions (A) and flow diagram for searching promoter motifs E-Box, D-Box and RORE for morning lncRNAs, evening lncRNAs and night lncRNAs, respectively (B). Supplementary Figure S2. Principal Component Analysis (PCA) of circadianly expressed zebrafish larval lncRNAs under the DD condition. All circadianly expressed larval lncRNAs (A), morning (CT0 and CT4) lncRNAs (B), evening (CT8 and CT12) lncRNAs (C), and night (CT16 and CT20) lncRNAs (D). Supplementary Figure S3. GO annotation and KEGG pathway analyses of zebrafish larval lncRNAs under the DD condition. GO annotation revealed the percentage of genes potentially involved in different biological processes (A), and KEGG pathways analysis of representative lncRNAs (B). Supplementary Figure S4. Principal Component Analysis (PCA) of circadianly expressed zebrafish larval lncRNAs under the LL condition. All circadianly expressed larval lncRNAs (A), morning (CT0 and CT4) lncRNAs (B), evening (CT8 and CT12) lncRNAs (C), and night (CT16 and CT20) lncRNAs (D). Supplementary Figure S5. GO annotation and KEGG pathway analyses of zebrafish larval lncRNAs under the LL condition. GO annotation revealed the percentage of genes potentially involved in different biological processes (A), and KEGG pathways analysis of representative lncRNAs (B). Supplementary Figure S6. Principal Component Analysis (PCA) of 30 larval lncRNAs coexpressed under both DD and LL conditions with expression profiles from the DD condition. All circadianly expressed larval lncRNAs (A), morning (CT0 and CT4) lncRNAs (B), evening (CT8 and CT12) lncRNAs (C), and night (CT16 and CT20) lncRNAs (D). Supplementary Figure S7. Expression profile analysis of morning (CT0 and CT4), evening (CT8 and CT12) and night (CT16, CT20) zebrafish larval lncRNAs coexpressed under both DD and LL conditions. Analysis of all 30 coexpressing larval lncRNAs with expression profiles from the LL condition (A-D): Heat map (A) and BioDare2 plots (B) of all 30 coexpressing larval lncRNAs, expression profiles (C) and BioDare2 plots (D) of representative lncRNAs. (E-H) Analysis of nine larval morning lncRNAs: Heat map (E) and BioDare2 plots (F) of nine larval morning lncRNAs, expression profiles (G) and BioDare2 plots (H) of larval morning representative lncRNAs. (I-L) Analysis of 14 larval evening lncRNAs: Heat map (I) and BioDare2 plots (J) of 14 larval evening lncRNAs, expression profiles (K) and BioDare2 plots (L) of larval evening representative lncRNAs. (M-P) Analysis of seven larval night lncRNAs: Heat map (M) and BioDare2 plots (N) of 7 larval night lncRNAs, expression profiles (O) and BioDare2 plots (P) of larval night representative lncRNAs. Supplementary Figure S8. Principal Component Analysis (PCA) of 30 circadianly expressed zebrafish larval lncRNAs under both DD and LL conditions with expression profiles from the LL condition. All circadianly expressed larval lncRNAs (A), morning lncRNAs (B), evening lncRNAs (C), and night lncRNAs (D). Supplementary Figure S9. GO annotation and KEGG pathway analyses of zebrafish larval lncRNAs coexpressed under both DD and LL conditions. GO annotation revealed the percentage of genes involved in different biological processes (A), and KEGG pathways analysis of representative lncRNAs (B). Supplementary Table S1. Expression profiles of zebrafish larval transcripts under the DD condition. Supplementary Table S2. Expression profiles of zebrafish larval transcripts under the LL condition. Supplementary Table S3. Zebrafish larval lncRNAs identified from the unannotated transcripts, their expression profiles and known identifiers from Gene Bank and Ensembl. Supplementary Table S4. Rhythmicity analysis of the expression profiles of zebrafish larval lncRNAs under the DD condition by MetaCycle. Supplementary Table S5. Circadianly expressed larval zebrafish lncRNAs under the DD condition with corresponding Gene Bank ID/Ensembl IDs and their classification into morning, evening, and night lncRNAs. Supplementary Table S6. Mapping of morning lncRNAs under the DD condition to Ensembl IDs. Supplementary Table S7. Mapping of evening lncRNAs under the DD condition to Ensembl IDs. Supplementary Table S8. Mapping of night lncRNAs under the DD condition to Ensembl IDs. Supplementary Table S9. Summary of promoter analysis of zebrafish larval morning, evening, and night lncRNAs under the DD condition. Supplementary Table S10. Promoter analysis of zebrafish larval morning lncRNAs under the DD condition with the Matlab program and the Find Individual Motif Occurrences (FIMO) tool. Supplementary Table S11. Promoter analysis of zebrafish larval evening lncRNAs under the DD condition with the Matlab program and the Find Individual Motif Occurrences (FIMO). Supplementary Table S12. Promoter analysis of zebrafish larval night lncRNAs under the DD condition with the Matlab program and the Find Individual Motif Occurrences (FIMO) tool. Supplementary Table S13. GO analysis of zebrafish larval morning, evening, and night lncRNAs under the DD condition. Supplementary Table S14. KEGG pathway analysis of zebrafish larval morning, evening, and night lncRNAs under DD condition. Supplementary Table S15. Rhythmicity analysis of the expression profiles of zebrafish larval lncRNAs under LL condition by MetaCycle. Supplementary Table S16. Circadianly expressed larval zebrafish lncRNAs under the LL condition with corresponding Gene Bank IDs/Ensembl IDs and their classification into morning, evening, and night lncRNAs. Supplementary Table S17. Mapping of morning lncRNAs under LL condition to Ensembl IDs. Supplementary Table S18. Mapping of evening lncRNAs under the LL condition to Ensembl IDs. Supplementary Table S19. Mapping of night lncRNAs under the LL condition to Ensembl IDs. Supplementary Table S20. Summary of promoter analysis of zebrafish larval morning, evening, and night lncRNAs under the LL condition. Supplementary Table S21. Promoter analysis of zebrafish larval morning lncRNAs under the LL condition with the Matlab program and the Find Individual Motif Occurrences (FIMO) tool. Supplementary Table S22. Promoter analysis of zebrafish larval evening lncRNAs under the LL condition with the Matlab program and the Find Individual Motif Occurrences (FIMO) tool. Supplementary Table S23. Promoter analysis of zebrafish larval night lncRNAs under the LL condition with the Matlab program and the Find Individual Motif Occurrences (FIMO) tool. Supplementary Table S24. GO analysis of zebrafish larval morning, evening, and night lncRNAs under the LL condition. Supplementary Table S25. KEGG pathway analysis of zebrafish larval morning, evening, and night lncRNAs under the LL condition. Supplementary Table S26. Zebrafish larval lncRNAs expressed circadianly only under the DD condition, only under the LL condition, and under both DD and LL conditions. Supplementary Table S27. Classification of zebrafish larval lncRNAs coexpressed under both DD and LL conditions into morning, evening, and night lncRNAs using expression profiles from DD condition. Supplementary Table S28. Classification of zebrafish larval lncRNAs coexpressed under both DD and LL conditions into morning, evening, and night lncRNAs using expression profiles from LL condition and comparison of BioDare2 phase values of lncRNAs under DD and LL conditions. Supplementary Table S29. 60 zebrafish larval lncRNAs under DD condition coexpressed in the zebrafish pineal gland. Supplementary Table S30. 39 zebrafish larval lncRNAs under the DD condition coexpressed in the zebrafish testis. Supplementary Table S31. Nine zebrafish larval lncRNAs under the DD condition coexpressed in both the zebrafish pineal gland and testis. Supplementary Table S32. 64 zebrafish larval lncRNAs under the LL condition coexpressed in the zebrafish pineal gland. Supplementary Table S33. 59 zebrafish larval lncRNAs under the LL condition coexpressed in the zebrafish testis. Supplementary Table S34. 12 zebrafish larval lncRNAs under the LL condition coexpressed in both the zebrafish pineal gland and testis. Supplementary Table S35. Peptides encoded by nine lncRNAs under the DD condition observed simultaneously in larvae, pineal gland and testis. Supplementary Table S36. Peptides encoded by 12 lncRNAs under the LL condition observed simultaneously in larvae, pineal gland and testis. Supplementary Table S37. Known domains in Protein Data Bank shared by peptides encoded by coexpressing lncRNAs simultaneously observed in zebrafish larvae (DD condition), testis and pineal gland. Supplementary Table S38. The known domain in Protein Data Bank shared by peptides encoded by coexpressing lncRNAs simultaneously observed in zebrafish larvae (LL condition), testis and pineal gland. Supplementary Table S39. Human orthologs of circadianly expressed zebrafish larval lncRNAs under DD condition and their classification into morning lncRNAs, evening lncRNAs, and night lncRNAs. Supplementary Table S40. The mouse ortholog of circadianly expressed zebrafish larval lncRNAs under the DD condition. Supplementary Table S41. Three-species conservation of circadianly expressed zebrafish larval lncRNAs under DD and LL conditions. Supplementary Table S42. Human orthologs of circadianly expressed zebrafish larval lncRNAs under the LL condition and their classification into morning lncRNAs, evening lncRNAs, and night lncRNAs. Supplementary Table S43. Mouse orthologs of circadianly expressed zebrafish larval lncRNAs under the LL condition. Supplementary Table S44. Peptides encoded by zebrafish larval lncRNAs (DD condition) conserved with mouse orthologs and human orthologs. Supplementary Table S45. Peptides encoded by human orthologs conserved with zebrafish larval lncRNAs (DD condition). Supplementary Table S46: Peptides encoded by mouse orthologs conserved with zebrafish larval lncRNAs (DD condition). Supplementary Table S47. The lncRNA-encoded peptide conserved among zebrafish larvae (DD condition), mice, and humans. Supplementary Table S48. Peptides encoded by zebrafish larval lncRNAs (LL condition) conserved with mice orthologs and human orthologs. Supplementary Table S49. Peptides encoded by human orthologs conserved with zebrafish larval lncRNAs (LL condition). Supplementary Table S50. Peptides encoded by mice orthologs conserved with zebrafish larval lncRNAs (LL condition).
