# Supplementary material for: Hundreds of LncRNAs Display Circadian Rhythmicity in Zebrafish Larvae
Source: Cells. 2021 Nov 15;10(11):3173. doi: 10.3390/cells10113173 (PMC8620895; doi:10.3390/cells10113173)
Supplement: Supplementary file 1 [file cells-10-03173-s001.zip › Supplementary Figures S1-S9.pdf]

Supplementary Figures

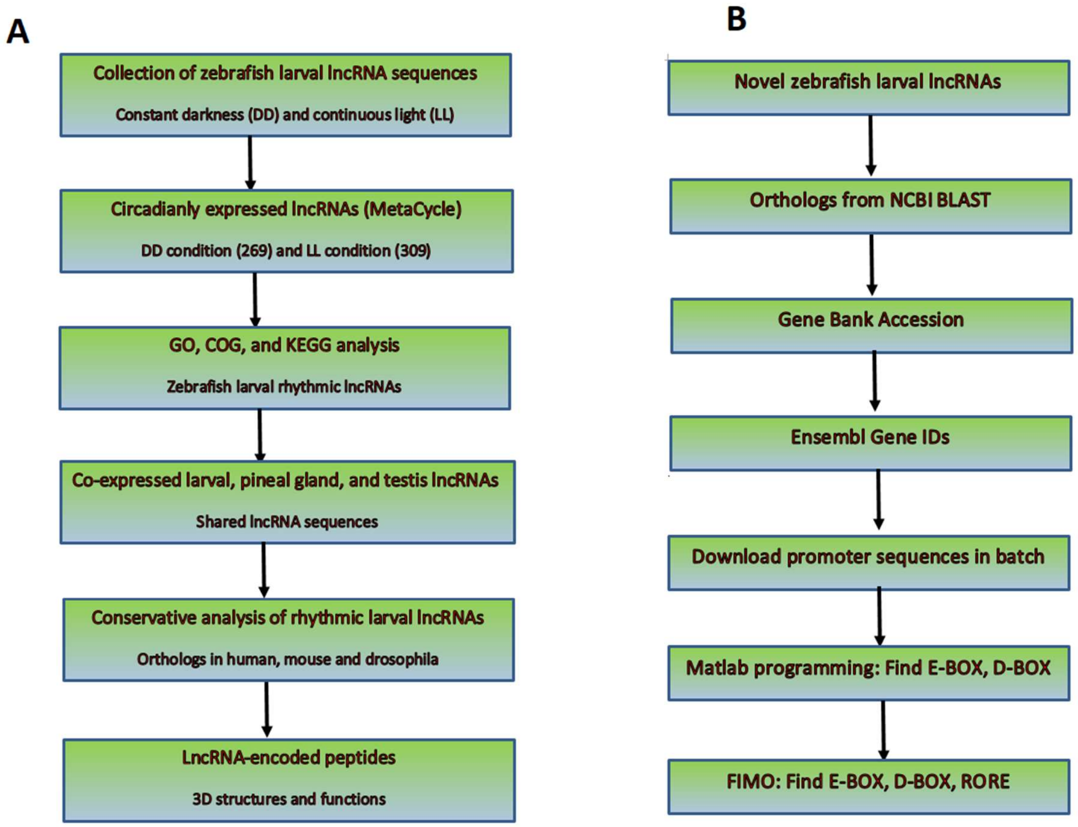

Figure S1. Research pipeline for investigating wild-type zebrafish larval lncRNAs under DD and LL conditions (**A**) and flow diagram for searching promoter motifs E-Box, D-Box and RORE for morning lncRNAs, evening lncRNAs and night lncRNAs, respectively (**B**).

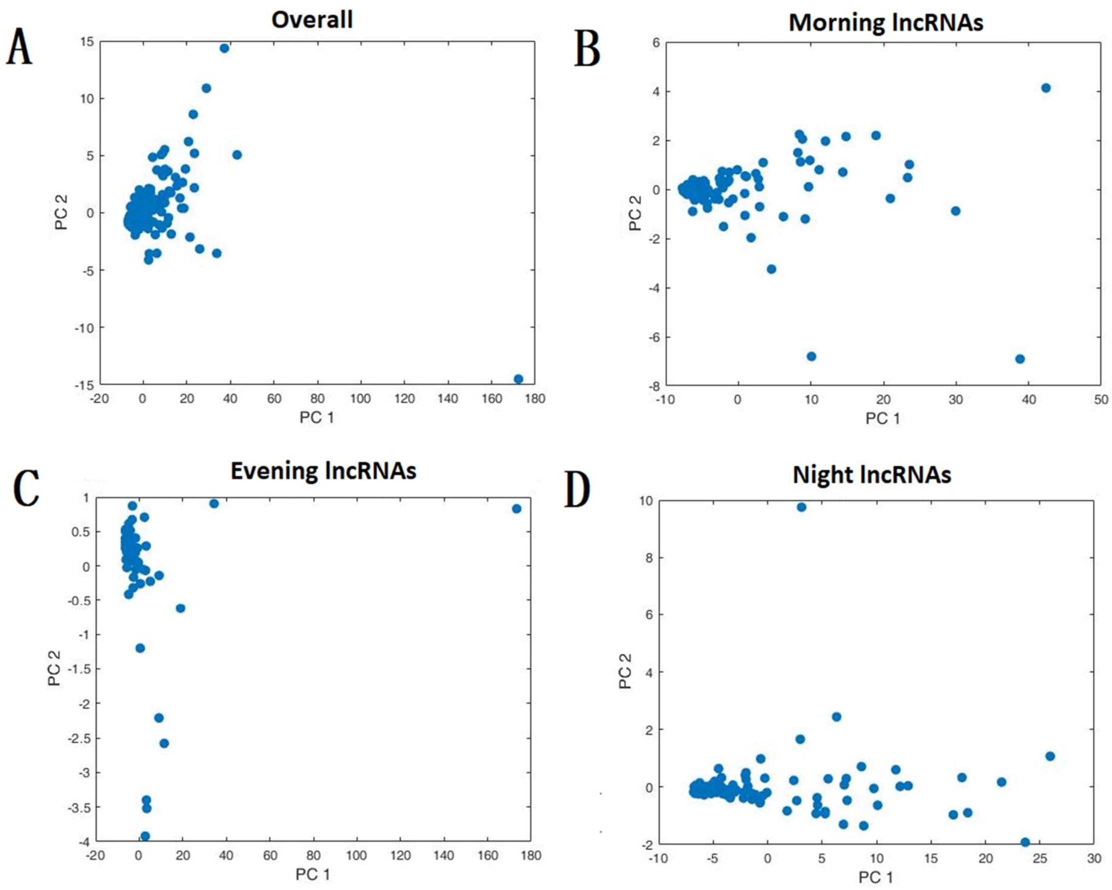

Figure S2. Principal Component Analysis (PCA) of circadianly expressed zebrafish larval lncRNAs under DD condition. All circadianly expressed larval lncRNAs (**A**), morning (CT0 and CT4) lncRNAs (**B**), evening (CT8 and CT12) lncRNAs (**C**), and night (CT16 and CT20) lncRNAs (**D**).

A

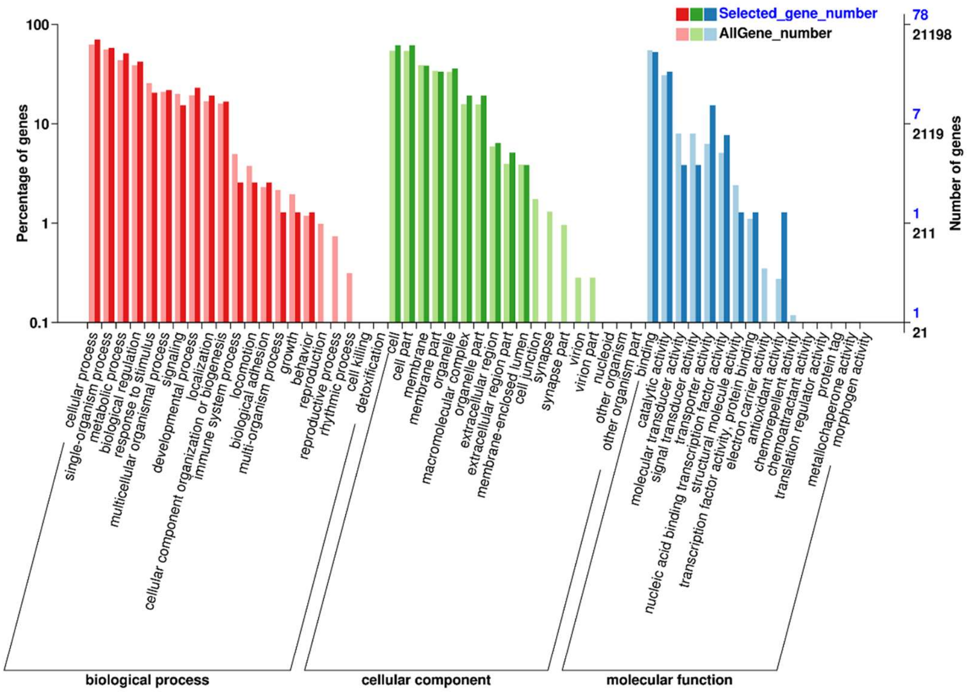

B

Danio_rerio_newGene_5385	Intracellular trafficking and secretion, GTPase activator activity, Arf GTPase activating protein, Endocytosis, ArfGap, Coiled coil, Complete proteome, Proteomics identification, Reference proteome
Danio_rerio_newGene_134	Transport, ion transport, potassium ion transport, sodium ion transport, cellular sodium ion homeostasis, potassium ion import, ATP hydrolysis coupled proton transport, cellular potassium ion homeostasis
Danio_rerio_newGene_9598	Intracellular trafficking and secretion, GTPase activator activity, Arf GTPase activating protein, ArfGap, Complete proteome, Reference proteome
Danio_rerio_newGene_20313	Response to yeast, transcription, DNA-templated, regulation of transcription, DNA-templated, response to bacterium, regulation of DNA methylation, transcription factor activity, sequence-specific DNA binding
Danio_rerio_newGene_6490	Peptidyl-proline hydroxylation to 3-hydroxy-L-proline, endoplasmic reticulum, Tetratricopeptide-like helical, Complete proteome, Proteomics identification, Reference proteome, Signal

Figure S3. GO annotation and KEGG pathway analyses of zebrafish larval lncRNAs under DD condition: GO annotation revealed the percentage of genes involved in different biological processes (**A**), and KEGG pathways analysis of representative lncRNAs (**B**).

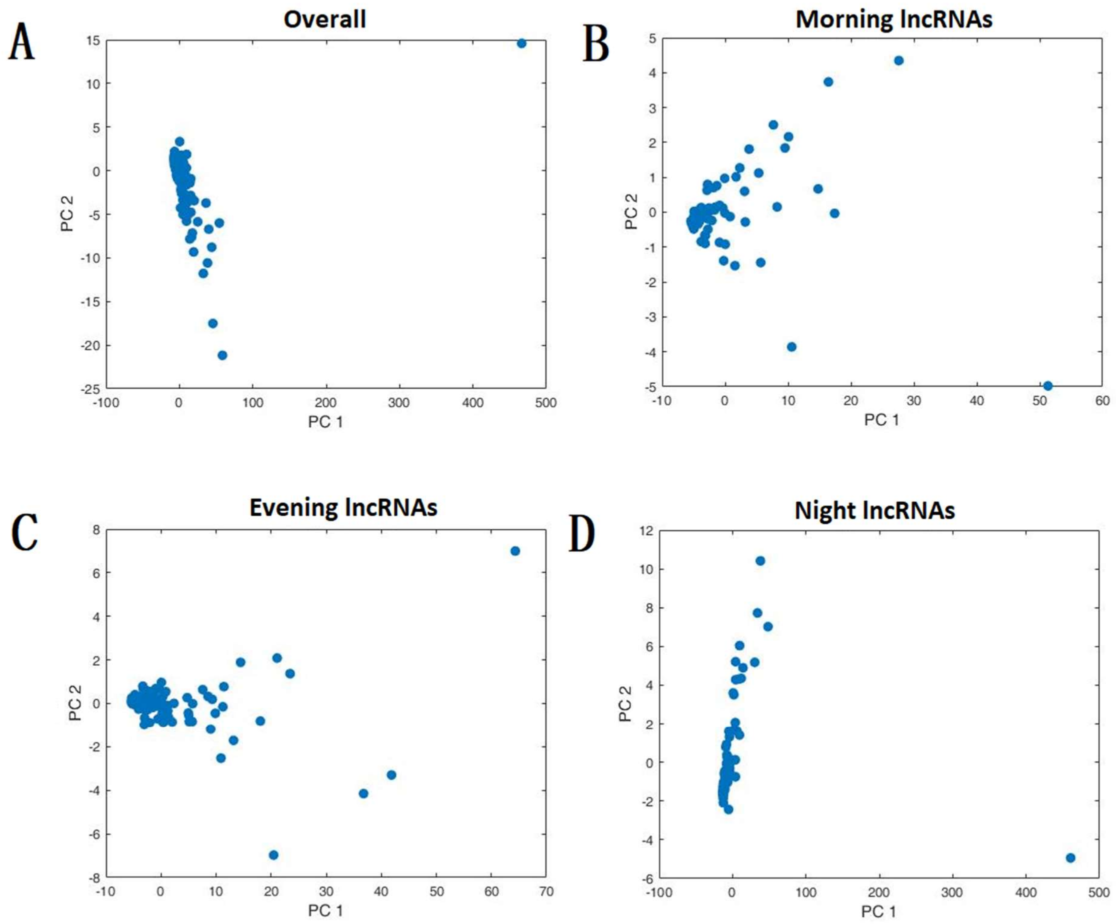

Figure S4. Principal Component Analysis (PCA) of circadianly expressed zebrafish larval lncRNAs under LL condition. All circadianly expressed larval lncRNAs (A), morning (CT0 and CT4) lncRNAs (B), evening (CT8 and CT12) lncRNAs (C), and night (CT16 and CT20) lncRNAs (D).

A

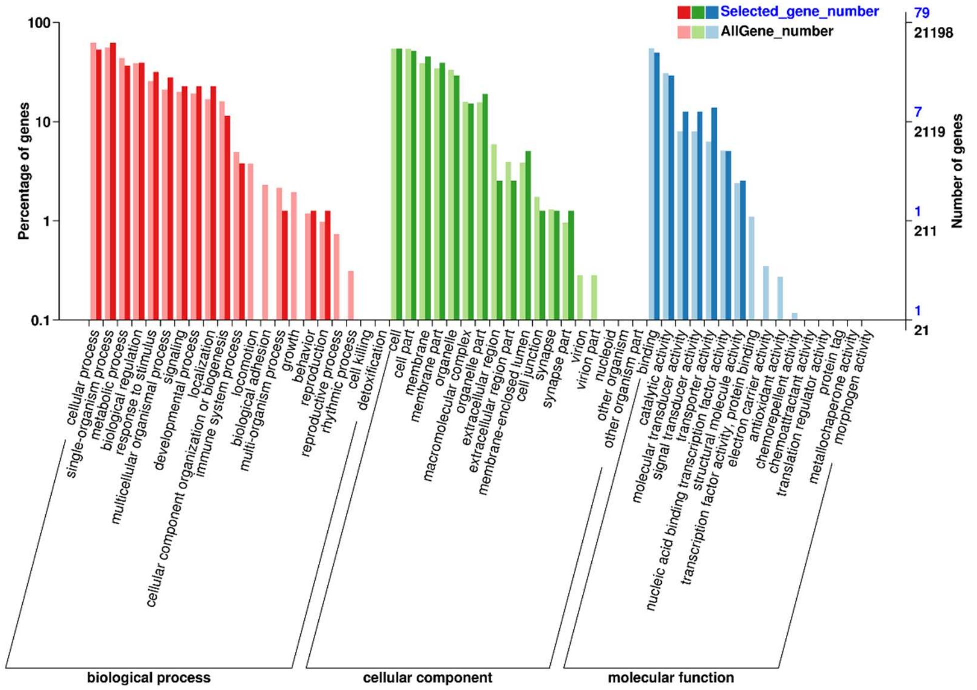

B

Danio_rerio_newGene_20758	Protein phosphorylation, regulation of bone mineralization, nucleus, protein kinase activity, protein serine/threonine kinase activity, ATP binding, phosphatase regulator activity, Protein kinase, catalytic domain
Danio_rerio_newGene_17099	Cation-transporting P-type ATPase, Cation-transporting P-type ATPase, N-terminal, Cation-transporting P-type ATPase, C-terminal, Calcium-transporting P-type ATPase, subfamily IIB, P-type ATPase, A domain, P-type ATPase, phosphorylation site
Danio_rerio_newGene_9866	Transport, intracellular protein transport, ER to Golgi vesicle-mediated transport, protein transport, COPII vesicle coat, zinc ion binding, von Willebrand factor, type A, Zinc finger, Sec23/Sec24-type, Sec23/Sec24, trunk domain, Sec23/Sec24, helical domain, Gelsolin domain, Sec23/Sec24 beta-sandwich
Danio_rerio_newGene_23775	Cell communication, signal transduction, cell surface receptor signaling pathway, G-protein coupled receptor signaling pathway, multicellular organism development, eye photoreceptor cell development, plasma membrane, membrane
Danio_rerio_newGene_13827	Type B pancreatic cell development, peptidyl-lysine modification to peptidyl-hypusine, exocrine pancreas development, deoxyhypusine biosynthetic process from spermidine, cytoplasm, deoxyhypusine synthase activity, Deoxyhypusine synthase, Complete proteome, Proteomics identification, Reference proteome

Figure S5. GO annotation and KEGG pathway analyses of zebrafish larval lncRNAs under LL condition: GO annotation revealed the percentage of genes involved in different biological processes (**A**), and KEGG pathways analysis of representative lncRNAs (**B**).

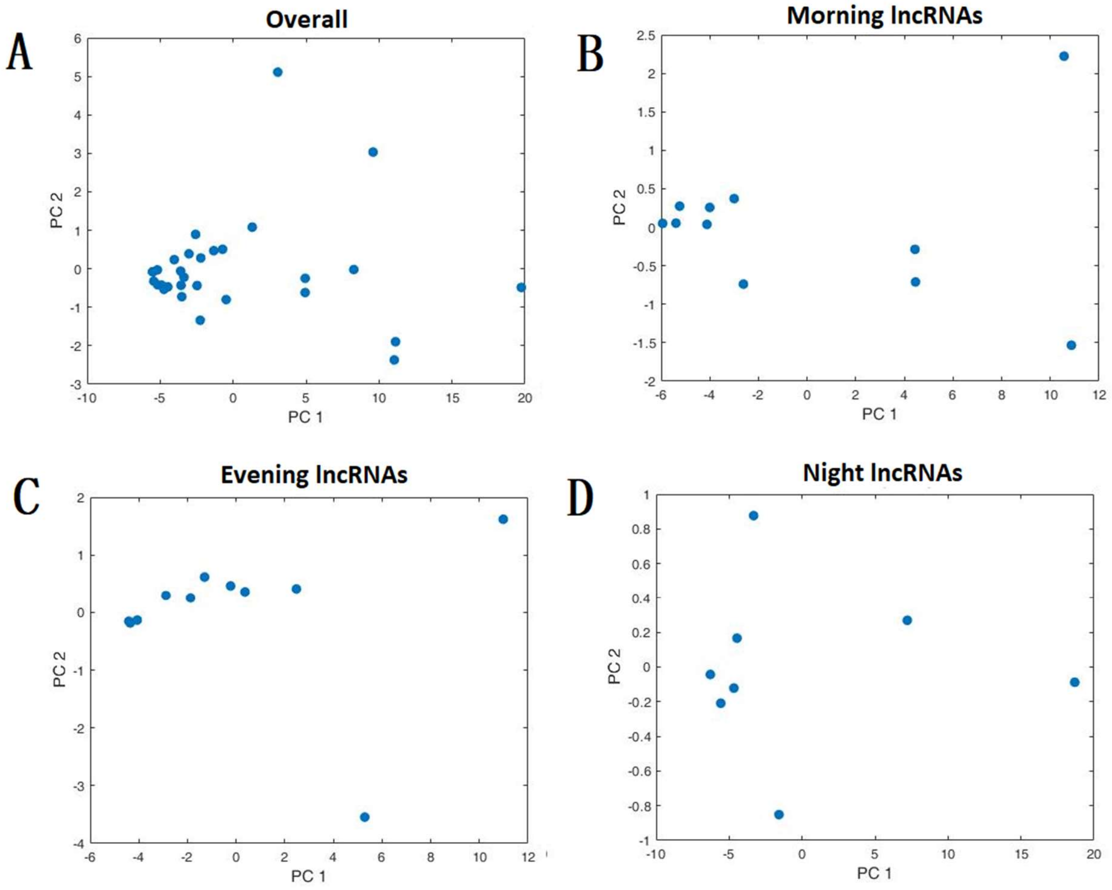

Figure S6. Principal Component Analysis (PCA) of 30 larval lncRNAs co-expressed under both DD and LL condition with expression profiles from DD condition. All circadianly expressed larval lncRNAs (**A**), morning (CT0 and CT4) lncRNAs (**B**), evening (CT8 and CT12) lncRNAs (**C**), and night (CT16 and CT20) lncRNAs (**D**).

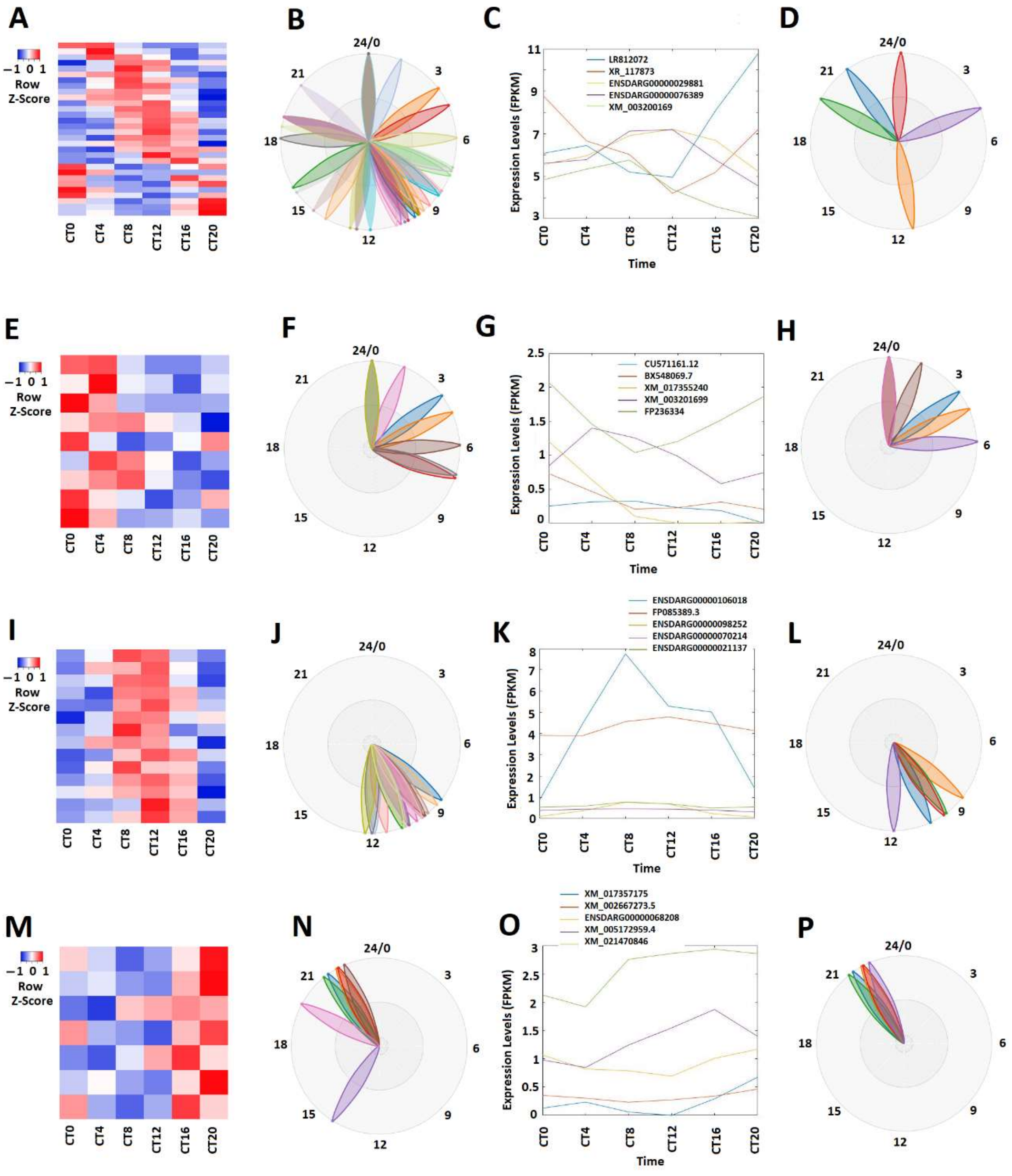

Figure S7. Expression profile analysis of morning (CT0 and CT4), evening (CT8 and CT12) and night (CT16, CT20) zebrafish larval lncRNAs co-expressed under both DD and LL conditions. Analysis of all 30 co-expressing larval lncRNAs with expression profiles from LL condition (**A**–**D**): Heat map (**A**) and BioDare2 plots (**B**) of all 30 co-expressing larval lncRNAs, expression profiles (**C**) and BioDare2 plots (**D**) of representative lncRNAs. (**E**–**H**) Analysis of 9 larval morning lncRNAs: Heat map (**E**) and BioDare2 plots (**F**) of 9 larval morning lncRNAs, expression profiles (**G**) and BioDare2 plots (**H**) of larval morning representative lncRNAs. (**I**–**L**) Analysis of 14 larval evening lncRNAs: Heat map (**I**) and BioDare2 plots (**J**) of 14 larval evening lncRNAs, expression profiles (**K**) and BioDare2 plots (**L**) of larval evening representative lncRNAs. (**M**–**P**) Analysis of 7 larval night lncRNAs: Heat map (**M**) and BioDare2 plots (**N**) of 7 larval night lncRNAs, expression profiles (**O**) and BioDare2 plots (**P**) of larval night representative lncRNAs.

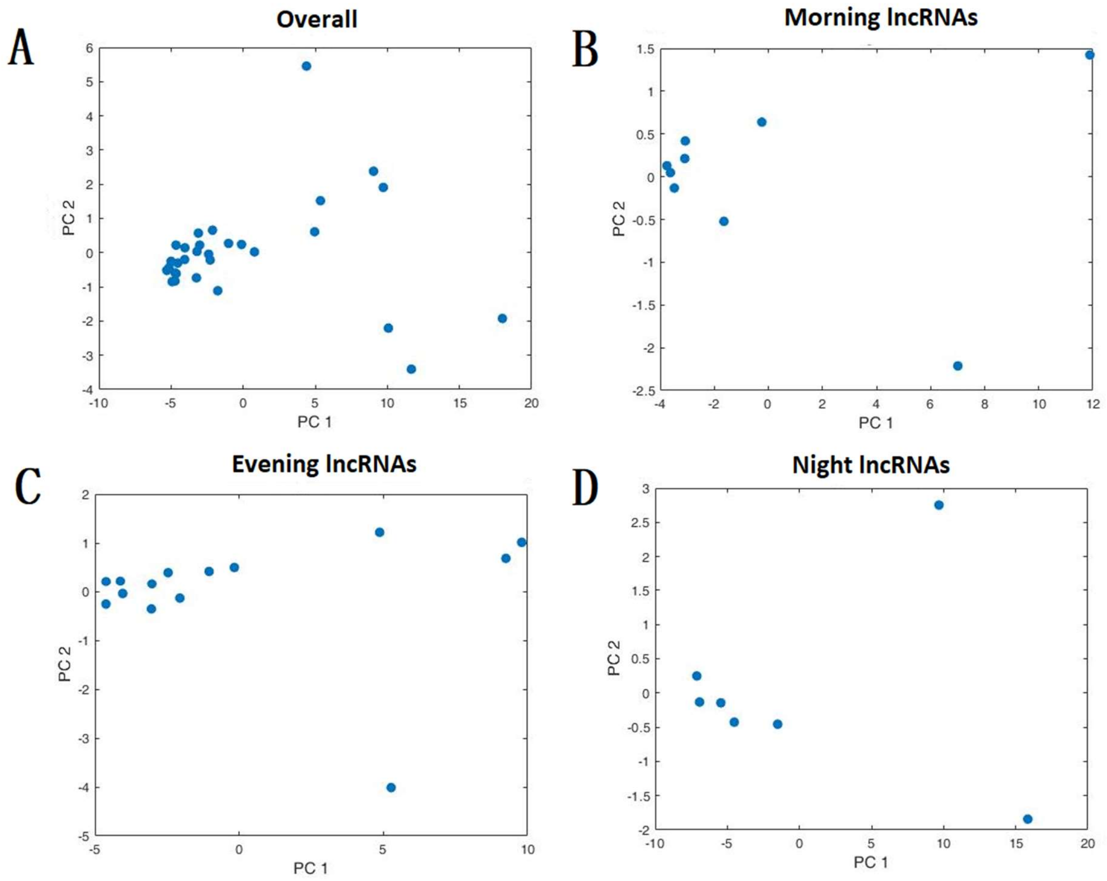

Figure S8. Principal Component Analysis (PCA) of 30 circadianly expressed zebrafish larval lncRNAs under both DD and LL condition with expression profiles from LL condition. All circadianly expressed larval lncRNAs (**A**), morning lncRNAs (**B**), evening lncRNAs (**C**), and night lncRNAs (**D**).

A

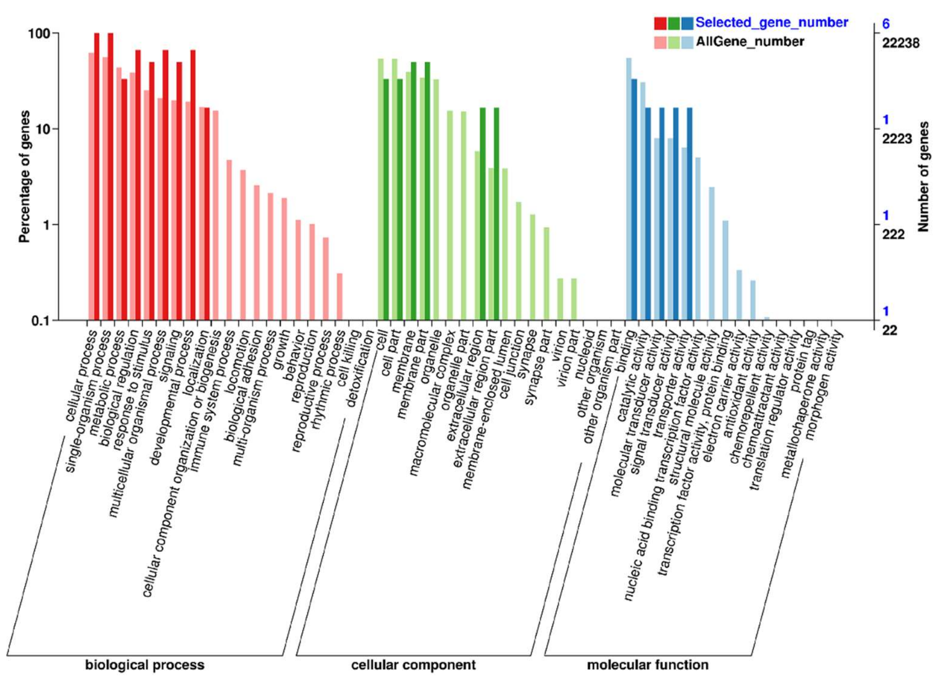

B

Danio_rerio_newGene_23775	Cell communication, signal transduction, cell surface receptor signaling pathway, G-protein coupled receptor signaling pathway, multicellular organism development, eye photoreceptor cell development, plasma membrane, membrane, integral component of membrane,
Danio_rerio_newGene_11483	C2 calcium-dependent membrane targeting, von Willebrand factor, type A, Copine, C2, VWA, Complete proteome, Reference proteome,
Danio_rerio_newGene_13827	Type B pancreatic cell development, peptidyl-lysine modification to peptidyl-hypusine, exocrine pancreas development, deoxyhypusine biosynthetic process from spermidine, cytoplasm, deoxyhypusine synthase activity, Deoxyhypusine synthase, Complete proteome, Proteomics identification, Reference proteome,
Danio_rerio_newGene_23948	Cation transport, chordate embryonic development, pronephros development, transmembrane transport, membrane, integral component of membrane, cation transmembrane transporter activity, SLC41 divalent cation transporters, integral membrane domain, Complete proteome, Membrane, Reference proteome, Transmembrane, Transmembrane helix
Danio_rerio_newGene_13547	miRNA mediated inhibition of translation, nucleotide binding, Nucleotide-binding, alpha-beta plait, Complete proteome, Reference proteome,

Figure S9. GO annotation and KEGG pathway analyses of zebrafish larval lncRNAs co-expressed under both DD and LL conditions: GO annotation revealed the percentage of genes involved in different biological processes (**A**), and KEGG pathways analysis of representative lncRNAs (**B**).
